# Isochromane-3,4-diones in Direct Vinylogous Aldol Reaction: Scope and Limitations

**DOI:** 10.3390/molecules31091508

**Published:** 2026-05-01

**Authors:** Sanam Gull Arshad, Mohammed Sadeq Mousavi, Consiglia Tedesco, Antonio Massa

**Affiliations:** Dipartimento di Chimica e Biologia “A. Zambelli”, Università Degli Studi di Salerno, 84084 Fisciano, SA, Italy; sarshad@unisa.it (S.G.A.); semousavi@unisa.it (M.S.M.); ctedesco@unisa.it (C.T.)

**Keywords:** Heterocycles, vinylogy, aldol reaction, lactones, isochromanones

## Abstract

Herein we report for the first time the use of isochromane-3,4-diones in a direct vinylogous aldol reaction with both aromatic and aliphatic aldehydes, affording the corresponding products in moderate to good yield and with very high diastereoselectivity in most cases. The reaction is enabled by a tertiary amine, which promotes the in situ formation of nucleophilic dienolate via γ-deprotonation of the α-ketoester functionality incorporated within the 3-isochromanone scaffold.

## 1. Introduction

Over the past few decades, vinylogous reactions have emerged as a powerful strategy for the construction of structurally complex building blocks [1,2,3,4,5]. Two main strategies have been pursued: the classical use of silyl dienolethers in indirect-type Mukayama reactions promoted by Lewis acids [1,2,3,4,5,6,7,8,9,10], and more recently, the in situ generation of dienols facilitated by weak bases under catalytic conditions [5,10,11,12,13,14,15,16,17]. Successful vinylogous aldol reactions are mostly associated with the formation of dienol derivatives from enals, while the use of enones as γ-selective pronucleophiles remains less explored [5]. Achieving γ-regioselectivity in reactions involving α,β-unsaturated ketones as pronucleophiles is particularly challenging. This is primarily due to the low electron density at the γ-position of the corresponding dienolates, which tends to favour non-vinylogous α-selective reactions. The presence of further enolizable α’ carbon is another drawback that can affect the selectivity [5]. Often activated ketones such as trifluoromethyl ketones or α-keto esters are employed as electrophilic partners [18,19,20]. Therefore, one of the main challenges in expanding the scope of this concept lies in the identification of novel vinylogous nucleophiles [5].

**Scheme 1:**
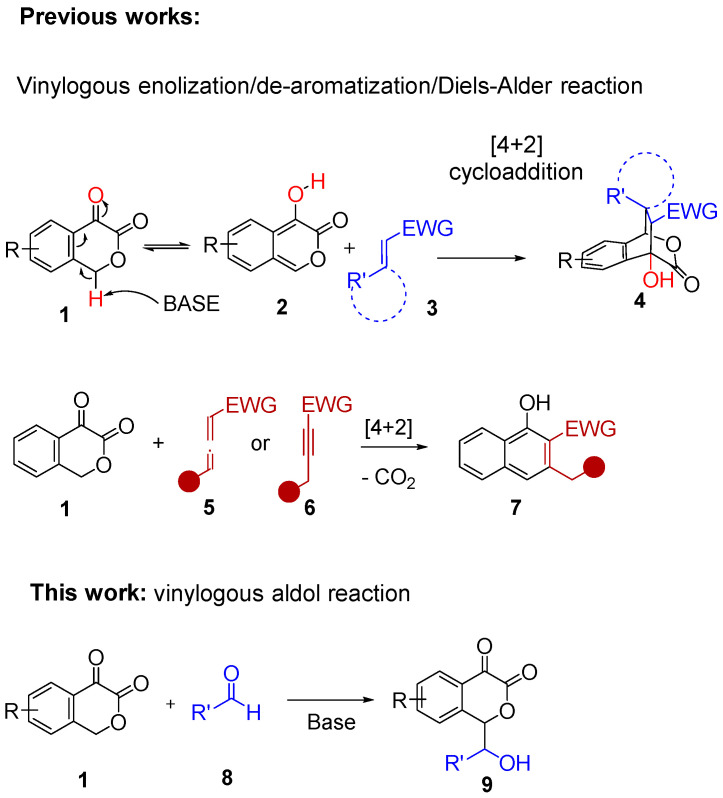
Previous studies and this work.

Recently, our group reported the use of isochroman-3,4-dione **1** in [4+2] cycloadditions to afford bridged lactone **4** analogues of natural products [21,22], or in decarboxylative cycloaddition/aromatization reactions to yield functionalized naphthols **7** [22]. Mildly basic conditions were required to promote the vinylogous enolization, enabling access to highly reactive diene species **2** that could react with electron-poor alkenes **3** [21,22], allenoates **5** [23] or alkynoates **6** [23]. Based on experimental evidence, we proposed that in all cases, the cycloadditions proceeded through concerted rather than stepwise pathways [21,22]. On the other hand, the in situ-generated dienol derived from isochromane-3,4-dione could also favour vinylogous γ-reactions when the electrophilic partner is less prone to undergo cycloaddition. This strategy would allow, selectively, the functionalization at 1 position of the 3-isochromanone, a transformation that has so far proven particularly challenging (Scheme 1).

## 2. Results and Discussion

**Figure 1:**
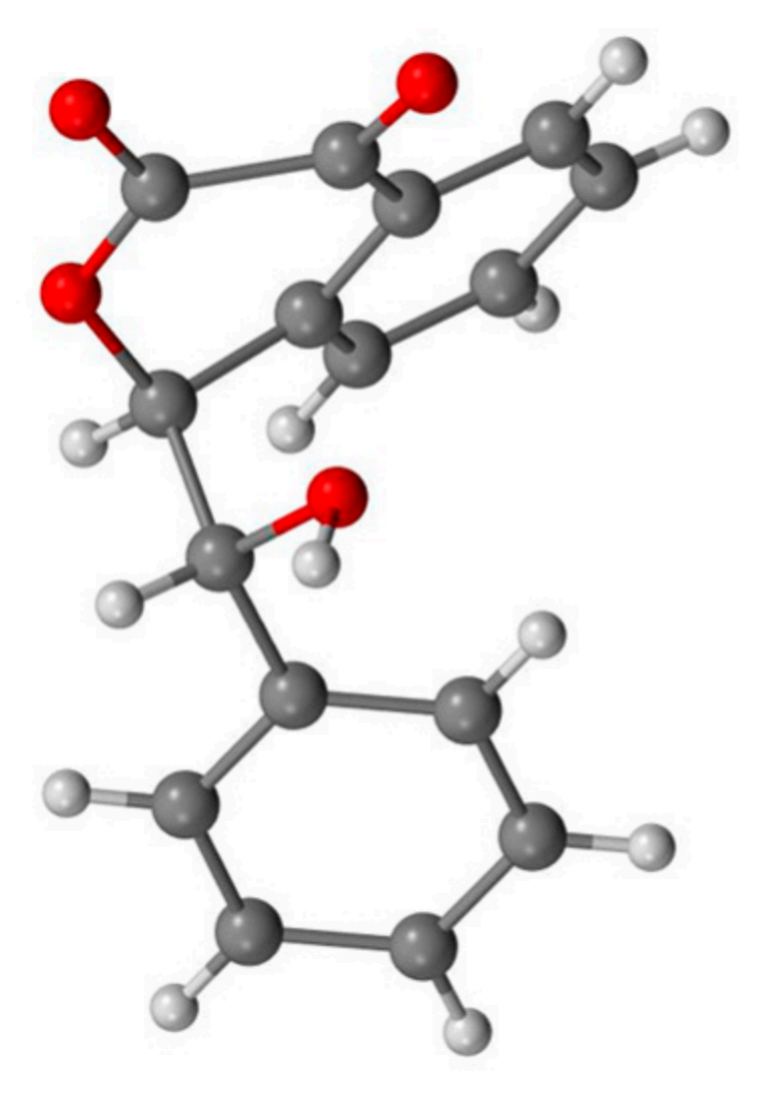
ORTEP drawing of compound **9a**. Ellipsoids are shown at 50% probability level (CCDC code: 2529279). Crystals of **9a** were obtained by slow evaporation of a solution of **9a** (5 mg) in a hexane/methanol mixture (1.5 mL, *v*/*v* = 2:1).

With this idea in mind, we started our investigation by reacting benzaldehyde **8a** with isochromane-3,4-dione **1** under basic conditions, under which the corresponding dienol species was generated in situ (Table 1). Optimization of the reaction parameters led to the formation of the vinylogous aldol adduct **9a**. The conditions were properly selected to favour the isolation of the pure product as a single diastereomer directly from the reaction mixture as an insoluble white solid in the presence of two equivalents of DIPEA and when the molar concentration of the limiting reagent was equal to or exceeded 0.4 M in DCM as the solvent (Entry 1). The structure of **9a** was also confirmed by X-ray structural analysis performed on single crystals (Figure 1), which allowed us to determine the relative configuration as (R*,S*).

This isolation process was facilitated by the absence of inorganic reagents and by the efficient removal of the organic base, unreacted starting materials, and by-products through an optimized protocol (see experimental procedure). Notably, this procedure eliminates the need for chromatography, which is considered a key advantage in pharmaceutical manufacturing, where avoiding chromatographic purification is highly desirable for API scale-up [24,25]. The reaction and the isolation protocol was also reproducible at 1 mmol scale with only a slight decrease in isolated yield of 55% and with similar high purity.

Analysis of the mother liquor composition proved challenging; however, following chromatographic purification, the overall isolated yield increased to 87% (Entry 2), and no other diastereomer was detected.

Further analysis of the reaction parameters highlighted that more diluted conditions, lower amounts of base or shorter reaction times gave a significantly lower conversion or decomposition products as detected by ^1^H NMR on the crude (Entries 3–5). The effect of concentration can be the result of the fact that the reaction under thermodynamic control does not display elevated conversion, and only when the solubility decreases is the equilibrium shifted toward the formation of the solid product according to the scheme of Table 1. More concentrated conditions led to the final products in higher yield but with lower purity (Entry 6). The use of CHCl_3_ led to similar results since we also observed low solubility of the final product in this solvent (Entry 7). With the aim of quantitatively generating the dienolate species, we also tried NaH as base in anhydrous DMSO, but we observed the formation of decomposition products (Entry 8). The use of other inorganic bases like K_2_CO_3_ or Li_2_CO_3_ in acetonitrile (ACN) also led to decomposition products (Entries 9 and 10).

We then analyzed the scope of the reaction, relying on similar low-solubility behaviour (Table 2). In most cases, the aldol products were obtained in moderate to good yields as single diastereomers via simple filtration. Their low solubility enabled straightforward purification by washing the obtained solid and avoiding chromatography, as previously described for **9a**.

In a few cases (**9f**, **9n–p**, **9t**), increased solubility prevented efficient precipitation, and the products were isolated in lower yields by chromatography. The origin of these solubility differences may be attributed to the *ortho* substitution pattern, which can hinder efficient crystal packing in **9f** and **9n**. A similar effect may be observed for **9o** and **9p**, likely due to the presence of a long aliphatic chain. Minor quantities of the opposite diastereomer were occasionally observed. The analysis of the mother liquors was challenging due to their complexity and was therefore not systematically pursued.

Analyzing the scope in detail, halogenated benzaldehydes afforded products **9b**–**9g** in good yields. If on one hand **9g** (from *o*-iodobenzaldehyde) was obtained in very good yield (85%), **9f** (from *o*-chlorobenzaldehyde) was more soluble and isolated in lower yield (46%) after chromatography as a mixture of diastereomers. Electron-withdrawing substituents (cyano, nitro, trifluoromethyl) provided comparable moderate yields (**9h**–**9j**). Isonicotinaldehyde (**9k**) and 2-naphthaldehyde (**9l**) were also well tolerated, although **9k** revealed very little solublility even in DMSO. While 4-methoxybenzaldehyde showed poor reactivity, 4- and 2-methylbenzaldehydes afforded products **9m** and **9n** respectively in acceptable yields. Aliphatic aldehydes (octanal and decanal) gave moderate yields of diastereomeric mixtures after chromatography (**9o**, **9p**). Substituted isocroman-3,4-diones with methoxy, bromo and nitro substituents delivered products **9q**–**9t** in moderate yields upon reaction with substituted benzaldehydes. Notably, 8-nitroisochromane-3,4-dione was reactive under these conditions, in contrast to previous Diels–Alder reports [21,22,23], furnishing **9t** as an inseparable diastereomeric mixture after chromatography.

Follow-up reactivity was extensively investigated in **9a** through oxidation reactions using various oxidants, including Dess–Martin periodinane, MnO_2_, and PCC, as well as dehydration with *p*-TSA and reduction processes. However, the low solubility of these compounds in common solvents, such as halogenated, aromatic, and oxygenated media, significantly limited their applicability.

Oxidation of the more soluble derivatives **9o** and **9p** was also explored, but these attempts predominantly resulted in decomposition products.

**Scheme 2:**
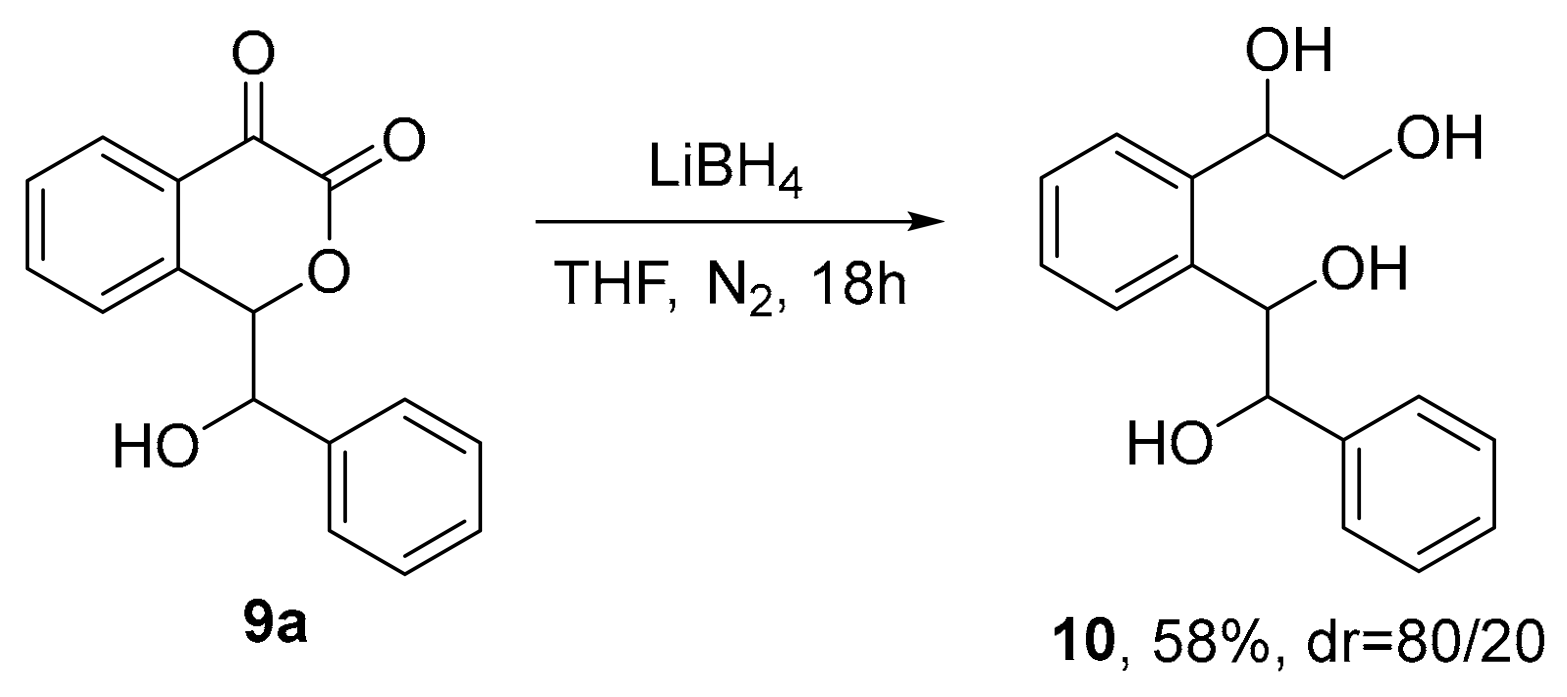
Reductive ring opening of **9a**.

Finally, the novel tetrol **10** with the pinacol motif, which is of great interest in further elaborations regarding the synthesis of bioactive compounds [26,27], was successfully synthesized via reductive ring opening of compound **9a**. Despite its limited solubility in THF, the reaction proceeded in the presence of LiBH_4_ to afford the product in moderate yield as a mixture of two diastereomers in an 80:20 ratio (Scheme 2).

## 3. Materials and Methods

Unless otherwise noted, all chemicals, reagents and solvents for the performed reactions are commercially available. 3-Isochromanone was purchased from Fluorochem. Isochromane-3,4-dione and substituted isochromane-3,4-diones were prepared according to the literature procedures [21,22,23]. All the reactions were monitored by thin-layer chromatography (TLC) on precoated silica gel plates (0.25 mm) and visualized by fluorescence quenching at 254 nm. Flash chromatography was carried out using neutral activated alumina (Merck, Darmstadt, Germany). The NMR spectra were recorded on Bruker DRX 600, 400, 300 and 250 MHz spectrometers (600 MHz, ^1^H, 150 MHz, ^13^C; 400 MHz, ^1^H, 100.6 MHz; ^13^C, 300 MHz, ^1^H, 75.5 MHz, ^13^C, 250 MHz, ^1^H, 62.5 MHz, ^13^C). The internal reference was set to the residual solvent signals (*δ*H 7.26 ppm, *δ*C 77.16 ppm for CDCl3, *δ*H 2.50 ppm, *δ*C 39.10 ppm for DMSO-d6). The ^13^C NMR spectra were recorded under broad-band proton decoupling. ^1^HNMR, ^13^CNMR and HRMS data are provided for all newly synthesized compounds. Copies of ^1^H and ^13^C NMR spectra for these compounds are included in the Supplementary Materials. The following abbreviations are used to indicate the multiplicity in NMR spectra: s—singlet, d—doublet, t—triplet, q—quartet, dd—doublet of doublets, m—multiplet, br s—broad signal. High-resolution mass spectra (HRMS) were acquired using a Bruker SolariX XR Fourier transform ion cyclotron resonance mass spectrometer (Bruker Daltonik GmbH, Bremen, Germany) equipped with a 7T refrigerated actively shielded superconducting magnet. For ionization of the samples, electrospray ionization (ESI) or MALDI was applied.

### 3.1. General Procedure for the Reaction of Dione with Benzaldehydes

To a solution of dione **1** (35 mg, 0.2 mmol, 1 equiv.) and DIPEA (25 mg, 0.2 mmol, 1 equiv.) in DCM (0.5 mL), benzaldehyde (0.3. mmol, 1.5 equiv.) was added and the mixture was stirred until a precipitate formed (3 days). To the suspension, 0.5 mL of pentane was added, the mixture was sonicated for 30 s, and the supernatant solution was removed. The solid was then washed another two times with a mixture of pentane/DCM (8:2 ratio) and filtered off.

**1-(hydroxy(phenyl)methyl)isochromane-3,4-dione (9a)**

Yield = 34 mg from solid isolation + 10 mg from chromatography (86). Hexane (0.5 mL) was added to suspension to remove nonpolar impurities, followed by a wash with hexane/DCM (8:2), resulting in a white solid. Single diastereomer. M.p. 197.5–198.8 °C. ^1^H NMR (400 MHz, DMSO-d_6_): δ 7.84 (d, *J* = 7.6 Hz, 1H), 7.43 (t, *J* = 7.5 Hz, 1H), 7.30 (t, *J* = 7.5 Hz, 1H), 7.15 (s, 3H), 6.92 (s, 2H), 6.79 (d, *J* = 4.4 Hz, 1H), 6.31(d, *J* = 7.5 Hz, 1H), 5.90 (s, 1H), 5.19 (s, 1H). ^13^C NMR (100 MHz, DMSO-d_6_): δ 174.8, 159.0, 139.3, 136.5, 133.1, 132.4, 129.2, 128.1, 127.9, 127.3, 126.5, 126.4, 83.1, 73.3. HRMS (MALDI-FT ICR): *m*/*z* calcd. for [C_16_H_12_KO_4_]^+^: 307.0367; found: 307.0368.

The reaction was scaled up at 1 mmol of **1a,** obtaining 147 mg of **9a** (55% yield).

**1-((4-bromophenyl)(hydroxy)methyl)isochromane-3,4-dione (9b)**

Yield: 42 mg (60%). Hexane (0.5 mL) was added to suspension to remove nonpolar impurities, followed by a wash with hexane/DCM (8:2), resulting in a white solid. Single diastereomer. M.p. 227.1–228.4 °C. ^1^H NMR (400 MHz, DMSO-d_6_): δ 7.91 (d, *J* = 7.8 Hz, 1H), 7.80 (d, *J* = 3.6 Hz, 2H), 7.55 (m, 3H), 7.39 (d, *J* = 8.2 Hz, 2H), 6.49 (d, *J* = 4.9 Hz, 1H), 5.82 (s, 1H),4.69 (d, *J* = 4.9 Hz, 1H). ^13^C NMR (150 MHz, DMSO-d_6_): δ 176.2, 160.1, 142.5, 141.9, 136.7, 133.31, 132.7, 130.9, 130.7, 128.4, 127.9, 122.5, 84.8, 75.1. HRMS (MALDI-FT ICR): *m*/*z* calcd. for [C1_6_H_11_^81^BrKO_4_]^+^: 386.9453; found: 386.9486.

**1-((4-chlorophenyl)(hydroxy)methyl)isochromane-3,4-dione (9c)**

Yield = 31 mg (52%). Hexane (0.5 mL) was added to suspension to remove nonpolar impurities, followed by a wash with hexane/DCM (8:2), resulting in a white solid. Single diastereomer. M.p. 186.4–187.4 °C. ^1^H NMR (300 MHz, DMSO-d_6_): δ 7.95 (d, *J* = 7.5 Hz, 1H), 7.84 (s, 2H), 7.63–7.57 (m, 1H), 7.55–7.41 (m, 4H), 6.56 (d, *J* = 4.5 Hz, 1H), 5.87 (s, 1H), 4.76 (d, *J* = 4.4 Hz, 1H). ^13^C NMR (100 MHz, DMSO-d_6_): δ 174.8, 158.7, 140.7, 140.6, 135.2, 132.5, 131.9, 129.4, 128.9, 128.4, 126.9, 126.5, 83.5, 73.6. HRMS (MALDI-FT ICR): *m*/*z* calcd. for [C_16_H_12_^35^ClO_4_]^+^: 303.0419; found: 303.0435.

**1-((4-fluorophenyl)(hydroxy)methyl)isochromane-3,4-dione (9d)**

Yield = 32 mg (56%). Hexane (0.5 mL) was added to suspension to remove nonpolar impurities, followed by a wash with hexane/DCM (8:2), resulting in a white solid. Single diastereomer. M.p. 199.8–200.5 °C. ^1^H NMR (300 MHz, DMSO-d_6_): δ 7.89 (d, *J* = 7.5 Hz, 1H), 7.51–7.38 (m, 2H), 7.07–6.93 (m, 5H), 6.40 (d, *J* = 7.5 Hz, 1H), 5.94 (s, 1H), 5.25 (s, 1H). ^13^C NMR (150 MHz, DMSO-d_6_): δ 175.6, 162.4 (*J*_C-F_ = 161.2), 159.7, 137.2, 136.4, 134.1, 133.3, 130.2, 129.6 (*J*_C-F_ = 5.4), 128.1, 127.4, 115.8 (*J*_C-F_ = 14.1), 83.7, 73.5. ^19^F{^1^H} NMR (376 MHz, DMSO-d_6_): δ 114.8. HRMS (MALDI-FT ICR): *m*/*z* calcd. for [C_16_H_12_FO_4_]^+^: 287.0715; found: 287.0729.

**1-((3-bromophenyl)(hydroxy)methyl)isochromane-3,4-dione (9e)**

Yield: 42 mg (67%). Hexane (0.5 mL) was added to suspension to remove nonpolar impurities, followed by a wash with hexane/DCM (8:2), resulting in a white solid. single diastereomer. M.p. 183.9–185.2 °C. ^1^H NMR (300 MHz, DMSO-d_6_): δ 7.96 (d, *J* = 7.8 Hz, 1H), 7.85–7.88 (m, 2H), 7.70 (s, 1H), 7.64–7.60 (m, 1H), 7.51 (d, 7.85 Hz, 2H), 7.37 (t, *J* = 7.8 Hz, 1H), 6.61 (d, *J* = 4.8 Hz 1H), 5.92 (s, 1H), 4.77 (d, *J* = 4.3 Hz 1H). ^13^C NMR (100 MHz, DMSO-d_6_): δ 174.8, 158.7, 144.5, 140.5, 135.2, 131.9, 130.7, 130.6, 129.8, 129.5, 126.9, 126.5, 126.1, 121.9, 83.3, 73.5. HRMS (MALDI-FT ICR): *m*/*z* calcd. for [C_16_H_11_^79^BrNaO_4_]^+^: 368.9733; found: 368.9744.

**1-((2-chlorophenyl)(hydroxy)methyl)isochromane-3,4-dione (9f)**

Yield: 28 mg (46%). Purification by flash chromatography on silica gel (Hexane/Ethyl acetate, 1:1) obtained as a white solid. Mixture of diastereomers (87/13). ^1^H NMR (300 MHz, DMSO-d_6_) δ 7.99 (d, *J* = 6.0 Hz, 1H, minor diastereomer), 7.92 (d, 1H, *J* = 9.0 Hz, major diastereomer), 7.87 (d, *J* = 9.0 Hz, 1H, minor diastereomer), 7.70–7.62 (m, 3H, minor diastereomer), 7.54–7.47 (m, 2H, major diastereomer), 7.41–7.36 (m, 1H, major diastereomer), 7.31–7.25 (m, 1H, major diastereomer), 7.14 (d, *J* = 6.0 Hz, 1H, major diastereomer), 7.05 (t, *J* = 7.7 Hz, 1H, major diastereomer), 6.75 (d, *J* = 5.4 Hz, 1H, minor diastereomer), 6.48–6.26 (m, 2H, major diastereomer), 5.90 (d, *J* = 3.0 Hz, 1H major diastereomer), 5.81 (s, 1H, minor diastereomer), 5.41 (m, *J* = 5.2, 3.1 Hz, 1H, major diastereomer), 4.95 (d, *J* = 5.4 Hz, 1H, minor diastereomer). ^13^C NMR (250 MHz, DMSO-d_6_) (major diastereomer only): δ 174.6, 158.7, 136.3. 135.9, 133.2, 132.5, 131.0, 130.0, 129.5, 129.3, 128.8, 127.4, 127.0, 126.6, 80.4, 70.8. HRMS (MALDI-FT ICR): *m*/*z* calcd. for [C_16_H_11_ClKO_4_]^+^: 340.9977; found: 341.0012.

**1-(hydroxy(2-iodophenyl)methyl)isochromane-3,4-dione (9g)**

Yield: 68 mg (85%). Hexane (0.5 mL) was added to suspension to remove nonpolar impurities, followed by a wash with hexane/DCM (8:2), resulting in a white solid. M.p. 173.3–174.1 °C. ^1^H NMR (400 MHz, DMSO-d_6_): δ 8.02 (d, *J* = 7.7 Hz, 1H), 7.93 (t, *J* = 8.0 Hz, 2H) 7.83 (d, *J* = 7.6 Hz, 1H), 7.69 (t, *J* = 7.6 Hz 1H), 7.50 (t, *J* = 7.5 Hz 1H), 7.42 (d, *J* = 7.6 Hz 1H), 7.14 (t, *J* = 7.2 Hz, 1H), 6.84 (d, *J* = 5.0 Hz, 1H), 5.83 (s, 1H), 4.73 (d, *J* = 4.8 Hz, 1H). ^13^C NMR (100 MHz, DMSO-d_6_): δ 174.2, 158.1, 141.8, 140.0, 138.7, 135.0, 131.3, 129.9, 129.2, 129.0, 128.1, 126.7, 124.9, 97.5, 80.8, 77.3. HRMS (MALDI-FT ICR): *m*/*z* calcd. for [C_16_H_11_INaO_4_]^+^: 416.9595; found: 416.9589.

**4-((3,4-dioxoisochroman-1-yl)(hydroxy)methyl)benzonitrile (9h)**

Yield: 28 mg (48%). Hexane (0.5 mL) was added to suspension to remove nonpolar impurities, followed by a wash with hexane/DCM (1:1), resulting in a white solid. M.p.221.5–228.7 °C. ^1^H NMR (400 MHz, DMSO-d_6_): δ 7.99 (d, *J* = 7.6 Hz, 1H), 7.91 (d, *J* = 9.1 Hz, 4H), 7.72 (d, *J* = 7.9 Hz, 3H), 7.65 (s, 1H), 6.71 (d, *J* = 5.2 Hz, 1H), 5.97 (s, 1H), 5.91 (s, 1H), 4.88 (d, *J* = 4.4 Hz, 1H). ^13^C NMR (63 MHz, DMSO-d_6_): δ 174.6, 158.5, 147.2, 140.2, 135.1, 132.3, 131.8, 129.4, 127.8, 126.8, 126.4, 119.2, 110.5, 83.0, 73.6. HRMS (MALDI-FT ICR): *m*/*z* calcd. for [C_17_H_11_KO_4_]^+^: 332.0320; found: 332.0334.

**1-hydroxy(4-nitrophenyl) methyl) isochromanone-3,4 dione (9i)**

Yield: 42 mg (67%). Hexane (0.5 mL) was added to suspension to remove nonpolar impurities, followed by a wash with hexane/DCM (8:2), resulting in a white solid. Single diastereomer. M.p. 204.4–205.9 °C. ^1^H NMR (400 MHz, DMSO-d_6_): δ 8.23 (d, *J* = 7.6 Hz, 2H), 7.92 (d, *J* = 7.6 Hz, 1H), 7.84–7.80 (m, 2H), 7.75 (d, *J =* 7.9 Hz, 2H), 7.58 (t, *J =* 7.2 Hz, 1H), 6.71 (d, *J* = 4.1 Hz, 1H), 5.94 (s, 1H), 4.88 (d, *J* = 3.8 Hz, 1H). ^13^C NMR (75 MHz, DMSO-d_6_): δ 174.7, 158.6, 149.3, 147.3, 140.2, 135.2, 131.9, 129.6, 128.1, 126.9, 126.5, 123.5, 83.1, 73.6. HRMS (MALDI-FT ICR): *m*/*z* calcd. for [C_16_H_11_KNO_6_]^+^: 352.0218; found: 352.0245.

**1-(hydroxy(4-(trifluoromethyl)phenyl)methyl)isochromane-3,4-dione (9j)**

Yield: 38 mg (52%). Hexane (0.5 mL) was added to suspension to remove nonpolar impurities, followed by a wash with hexane/DCM (8:2), resulting in a white solid. Single diastereomer. M.p. 166.5–168.7 °C. ^1^H NMR (300 MHz, DMSO-d_6_): δ 7.98 (d, *J* = 7.7 Hz, 1H), 7.9 (d, *J* = 5.2 Hz, 2H), 7.81–7.68 (m, 4H), 7.68–7.58 (m, 1H), 6.66 (d, *J* = 5.1 Hz, 1H), 5.9 (s, 1H), 4.86 (d, *J* = 4.2 Hz, 1H). ^13^C NMR (150 MHz, DMSO-d_6_): δ 175.5, 159.3, 147.1, 141.1, 136.0, 132.7, 130.3, 129.4, 129.1, 128.5, 127.7, 127.4, 127.2, 126.1 (q, *J* = 3.1 Hz), 84.0, 74.5. ^19^F{^1^H} NMR (376 MHz, DMSO-d_6_): δ 60.7. HRMS (MALDI-FT ICR): *m*/*z* calcd. for [C_17_H_12_F_3_O_4_]^+^: 337.0683; found: 337.0691.

**1-(hydroxy(pyridin-4-yl)methyl)isochromane-3,4-dione (9k)**

Yield: 46 mg (86%). Hexane (0.5 mL) was added to suspension to remove nonpolar impurities, followed by a wash with hexane/DCM (8:2), resulting in a white solid. Single diastereomer. M.p.: 212.3–213.8 °C. ^1^H NMR (400 MHz, MeOD-d_4_/DMSO-d_6_): δ 8.60 (d, *J* = 4.3 Hz, 2H), 7.99 (d, *J* = 7.8 Hz, 1H) 7.87 (d, *J* = 3.5 Hz, 2H), 7.66–7.63 (m, 1H), 7.53 (d, *J* = 4.6 Hz, 2H), 5.98 (s, 1H), 4.81 (s, 1H). ^13^C NMR (100 MHz, MeOD-d_4_/DMSO-d_6_): δ 172.4, 158.5, 150.3, 149.6, 140.2, 135.2, 131.9, 129.4, 126.9, 126.4, 122.0, 83.0, 73.1. HRMS (MALDI-FT ICR): *m*/*z* calcd. for [C_15_H_11_NNaO_4_]^+^: 290.0787; found: 290.0798.

**1-(hydroxy(naphthalen-2-yl)methyl)isochromane-3,4-dione (9l)**

Yield: 43 mg (68%). Hexane (0.5 mL) was added to suspension to remove nonpolar impurities, followed by a wash with hexane/DCM (8:2), resulting in a white solid. Single diastereomer. M.p. 202.2–203.7 °C. ^1^H NMR (300 MHz, MeOD-d_4_): δ 7.98–7.85 (m, 7H), 7.71–7.61 (m, 2H), 7.55–7.49 (m, 2H), 6.65 (d, *J* = 4.4 Hz 1H), 6.03 (s, 1H), 4.94 (d, *J* = 3.9 Hz, 1H). ^13^C NMR (100 MHz, MeOD-d_4_): δ 174.9, 158.8, 140.8, 139.2, 135.2, 133.1, 132.9, 131.9, 129.4, 128.3, 128.0, 127.9, 126.9, 126.7, 126.5, 126.4, 125.7, 125.2, 83.6, 74.4. HRMS (MALDI-FT ICR): *m*/*z* calcd. for [C_20_H_14_KO_4_]^+^: 357.0524; found: 357.0534.

**1-(hydroxy(*p*-tolyl) methyl) isochromanone-3,4 dione (9m)**

Yield: 26 mg (46%). Hexane (0.5 mL) was added to suspension to remove nonpolar impurities, followed by a wash with hexane/DCM (8:2), resulting in a white solid. Single diastereomer. M.p. 206.5–207.7 °C. ^1^H NMR (600 MHz, DMSO-d_6_): δ 7.88 (d, *J* = 7.7 Hz, 1H), 7.80–7.75 (m, 2H), 7.57–7.52 (m, 1H), 7.28 (d, *J* = 8.3 Hz, 2H), 7.12 (d, *J* = 7.8 Hz, 2H), 6.32 (d, *J* = 4.7 Hz, 1H), 5.7 (s, 1H), 4.63 (d, *J* = 4.7 Hz, 1H), 2.24 (s, 3H). ^13^C NMR (150 MHz, DMSO-d_6_): δ 176.2, 160.2, 142.2, 133.9, 138.5, 136.6, 133.3, 130.8, 130.3, 128.4, 128.2, 127.9, 85.1, 75.6, 22.6. HRMS (MALDI-FT ICR): *m*/*z* calcd. for [C_17_H_15_O_4_]^+^: 283.0965; found: 283.0965.

**1-(hydroxy(*o*-tolyl)methyl)isochromane-3,4-dione (9n)**

Yield: 23 mg (41%). Purification by flash chromatography on silica gel (Hexane/Ethyl acetate, 8:2). Single diastereomer, obtained as a viscous oil. ^1^H NMR (300 MHz, CDCl_3_): δ 8.09 (d, *J* = 7.7 Hz, 1H), 7.49 (t, *J* = 7.6 Hz,1H) 7.33 (t, *J* = 7.5 Hz, 1H), 7.18 (d, *J* = 3.9 Hz, 2H), 6.97–6.92 (m, 1H), 6.52 (d, *J* = 7.8 Hz 1H), 6.29 (d, *J* = 7.7 Hz, 1H), 5.68 (s, 1H), 5.63 (s, 1H), 3.88 (s, 3H). ^13^C NMR (75 MHz, CDCl_3_): δ 174.3, 158.9, 135.4, 135.1 132.9, 132.8, 132.3, 130.2, 129.1, 128.2, 126.6, 125.8, 81.7, 72.0, 18.8. HRMS (MALDI-FT ICR): *m*/*z* calcd. for [C_17_H_15_O_4_]^+^: 283.0965; found: 283.0981.

**1-(1-hydroxyoctyl)isochromane-3,4-dione (9o)**

Yield: 38 mg (65%). Mixture of diastereomers (dr = 67/33). Purification by flash chromatography on silica gel (Hexane/Ethyl acetate, 8:2) afforded as a white solid, dr = 2/1. ^1^H NMR (400 MHz, DMSO-d_6_): δ 7.92 (d, *J* = 7.63 Hz, 1H, major diastereomer), 7.78 (m, 1H, mixture of diastereomers), 7.60 (m, 1H, mixture of diastereomers), 7.52 (d, *J* = 7.6 Hz 1H, major diastereomer), 6.03 (d, *J* = 6 Hz, 1H, major diastereomer), 5.73 (s, 1H, mixture of diastereomers), 5.66 (d, *J* = 5.2 Hz, 1H, minor diastereomer), 3.88 (s, major diastereomer), 3.50 (s, minor diastereomer), 1.27–1.29 (m, 12H, mixture of diastereomers), 0.86–0.82 (m, 3H, mixture of diastereomers). ^13^C NMR (100 MHz, DMSO-d_6_): δ 175.0, 174.9, 159.2, 158.9, 141.5, 137.8, 135.2, 134.1, 132.5, 131.7, 129.4, 129.1, 127.4, 127.0, 126.8, 126.0, 82.3, 82.2, 73.0, 71.5, 34.3, 33.0, 31.7, 31.6, 29.4, 29.2, 29.1, 29.0, 25.8, 25.7, 22.6, 22.5, 14.3. HRMS (MALDI-FT ICR): *m*/*z* calcd. for [C_17_H_22_KO_4_]^+^: 329.1150; found: 329.1188.

**1-(1hydroxydecyl) isochromanone-3,4-dione (9p)**

Yield: 35 mg (55%). Purification by flash chromatography on silica gel (Hexane/Ethyl acetate, 8:2) mixture of diastereomers (dr = 67/33), resulting in a white solid. ^1^H NMR (300 MHz, DMSO-d_6_): δ 7.90 (d, *J* = 7.8 Hz, 1H+1H, mixture of diastereomers), 7.75 (t, *J* = 6.9 Hz, 1H+1H, mixture of diastereomers), 7.58 (t, *J* = 7.0 Hz, 1H+1H, mixture of diastereomers), 7.50 (d, *J* = 7.9 Hz, 1H+1H, mixture of diastereomers), 6.02 (d, *J* = 6.3 Hz, 1H, major diastereomer), 5.70 (s, 1H+1H, mixture of diastereomers), 5.65 (d, *J* = 5.2 Hz, 1H, minor diastereomer), 3.85 (m, 1H major diastereomer), 3.46 (m, 1H, minor diastereomer), 1.21 (d, *J* = 13.7 Hz, 16H+8H, mixture of diastereomers), 0.83 (t, *J* = 11.1 Hz, 3H+3H, mixture of diastereomers). ^13^C NMR (63 MHz, DMSO-d_6_): δ 174.9, 174.8, 159.1, 158.9, 141.4, 137.6, 135.0, 133.9, 132.4, 131.6, 129.3, 128.9, 127.2, 126.8, 126.6, 125.9, 82.2, 72.9, 71.4, 34.2, 32.8, 31.6, 29.2, 28.8, 25.6, 22.4, 14.2. HRMS (MALDI-FT ICR): *m*/*z* calcd. for [C_19_H_26_NaO_4_]^+^: 341.1723; found: 341.1740.

**1-(hydroxy(2-ioddophenyl)methyl)-6-methoxyisochromane-3,4-dione (9q)**

Yield = 44 mg (60%). Hexane (0.5 mL) was added to suspension to remove nonpolar impurities, followed by a wash with hexane/DCM (8:2), resulting in a white solid (Single diastereomer). M.p. 190.6–192.1 °C. ^1^H NMR (300 MHz, DMSO-d_6_): δ 7.91 (d, *J* = 7.9 Hz 1H), 7.71 (d, *J* = 8.5 Hz 1H), 7.50–7.37 (m, 4H), 7.14–7.08 (m, 1H), 6.78 (d, *J* = 4.9 Hz 1H), 5.76 (s, 1H), 4.72 (d, *J* = 4.3 Hz 1H), 3.87 (s, 3H). ^13^C NMR (63 MHz, DMSO-d_6_): δ 174.4, 159.9, 158.2, 142.2, 139.2, 133.1, 132.8, 130.3, 129.2, 128.4, 127.1, 132.2, 109.4, 97.9, 80.9, 77.5, 56.2. HRMS (MALDI-FT ICR): *m*/*z* calcd. for [C_17_H_13_INaO_5_]^+^: 446.9700; found: 446.9723.

**7-bromo-1-(hydroxy(4-nitrophenyl) methyl)isochromanone (9r)**

Yield: 42 mg (53%). Hexane (0.5 mL) was added to suspension to remove nonpolar impurities, followed by a wash with hexane/DCM (8:2), resulting in a white solid. Mixture of diastereomers (dr = 93/7). ^1^H NMR (300 MHz, DMSO-d_6_): δ 8.41 (d, *J* = 8.5 Hz, 1H minor diastereomer), 8.36–8.19 (m, 3H, major diastereomer), 8.20–8.07 (m, 2H, minor diastereomer), 7.96–7.74 (m, 4H, major diastereomer), 7.69 (d, *J* = 8.3 Hz, 1H, minor diastereomer), 7.30 (t, *J* = 8.3 Hz, 3H, minor diastereomer), 6.78 (d, *J* = 5.3 Hz, 1H, major diastereomer), 6.54 (d, *J* = 1.7 Hz, 1H, minor diastereomer), 6.04 (d, *J* = 3.4 Hz, 1H, minor diastereomer), 5.96 (s, 1H, major diastereomer), 5.44 (s, 1H, minor diastereomer), 4.98 (d, *J* = 5.2 Hz, 1H, major diastereomer). ^13^C NMR (100 MHz, DMSO-d_6_): δ 174.1_(major)_, 158.4_(minor)_, 158.1_(major)_, 149.1_(major)_, 147.4_(major)_, 147.2_(minor)_, 141.1_(major)_, 137.8_(minor)_, 132.7_(major)_, 132.6_(minor)_, 131.8_(minor)_, 131.1_(major)_, 130.0_(minor)_, 129.5_(major)_, 129.4_(major)_, 129.1_(major)_, 128.8_(minor)_, 128.2_(major)_, 128.0_(minor)_, 127.6_(minor)_, 123.6_(major)_, 123.4_(minor)_, 82.5_(major)_, 81.5_(minor)_, 73.4_(major)_, 72.7_(minor)_. HRMS (MALDI-FT ICR): *m*/*z* calcd. for [C_16_H_10_^81^BrNNaO_6_]^+^: 415.9565; found: 415.9589.

**1-(hydroxy(4-nitrophenyl)methyl)-6-methoxyisochromanone-3,4-dione (9s)**

Yield: 36 mg (52%). Hexane (0.5 mL) was added to suspension to remove nonpolar impurities, followed by a wash with hexane/DCM (8:2), resulting in a white solid. Single diastereomer. M.p. 198.6–199.1 °C. ^1^H NMR (400 MHz, DMSO-d_6_): δ 8.30 (d, *J* = 8.6 Hz, 1H), 7.86–7.79 (m, 3H) 7.50–7.47 (m, 1H), 7.42 (d, *J* = 2.3 Hz 1H), 6.72 (d, *J* = 4.3 Hz 1H), 5.92 (s, 1H), 4.90 (d, *J* = 2.7 Hz, 1H), 3.89 (s, 3H). ^13^C NMR (100 MHz, DMSO-d_6_): δ 174.4, 159.9, 158.3, 149.3, 147.2, 133.0, 128.1, 123.5, 123.0, 109.1, 82.8, 73.5, 56.0. HRMS (MALDI-FT ICR): *m*/*z* calcd. for [C_17_H_13_NNaO_7_]^+^: 366.0585; found: 366.0592.

**1-((3-bromophenyl)(hydroxy)methyl)-8-nitroisochromanone-3,4-dione (9t)**

Yield: 30 mg (55%) Purification by flash chromatography on silica gel (Hexane/Ethyl acetate, 8:2). Mixture of diastereomers (dr = 71/29), obtained as a viscous liquid. ^1^H NMR (400 MHz, DMSO-d_6_): δ 8.65 (d, *J* = 8.1 Hz, 1H, major diastereomer), 8.54 (d, *J* = 8.1 Hz, 1H, minor diastereomer), 8.32 (d, *J* = 7.5 Hz, 1H, major diastereomer), 8.08 (d, *J* = 7.6 Hz, 1H, minor diastereomer), 7.96 (t, *J* = 7.8 Hz, 1H, major diastereomer), 7.85 (t, *J* = 7.8 Hz, 1H, minor diastereomer), 7.64 (s, 1H, major diastereomer), 7.58–7.46 (m, 2H, major diastereomer), 7.41 (t, *J* = 7.8 Hz, 1H, major diastereomer), 7.35 (d, *J* = 7.9 Hz, 1H, minor diastereomer), 7.08 (t, *J* = 7.8 Hz, 1H, minor diastereomer), 6.97 (d, *J* = 7.8 Hz, 1H, minor diastereomer), 6.40 (d, *J* = 4.4 Hz, 1H, major diastereomer), 6.37 (d, *J* = 3.0 Hz, 1H, minor diastereomer), 5.78 (d, *J* = 19.0 Hz, 1H, minor diastereomer), 5.66 (d, *J* = 6.0 Hz, 1H, major diastereomer), 5.36 (d, *J* = 6.0 Hz, 1H, major diastereomer), 5.25 (t, *J* = 4.1 Hz, 1H, minor diastereomer). ^13^C NMR (100 MHz, DMSO-d_6_): δ 168.5_(major)_, 168.0_(minor)_, 144.7, 144.3, 143.9, 142.3, 141.6, 141.0, 132.3, 132.2, 131.8, 131.5, 130.9, 130.8, 130.6, 130.3, 130.1, 130.0, 129.9, 129.6, 129.3, 126.4, 125.6, 121.9, 121.4, 85.3_(major)_, 84.7_(minor)_, 72.6_(minor)_, 68.8_(major)_. HRMS (MALDI-FT ICR): *m*/*z* calcd. for [C_16_H_10_^79^BrNNaO_6_]^+^: 413.9584; found: 413.9597.

### 3.2. Synthesis of 1-(2-(1,2-Dihydroxyethyl) phenyl)-2-phenylethane-1,2-diol

LiBH_4_ (0.3 mmol, 160 µL of 2M solution in THF, 3 equiv.) was added dropwise to a solution of 1-(hydroxy(phenyl)methyl)isochromane-3,4-dione (0.2 mmol, 54 mg, 1 equiv.) in the anhydrous THF (1 mL) under nitrogen atmosphere at 0 °C for 1 h, and the reaction mixture was allowed to stir at room temperature overnight. Then, the mixture was diluted with water (200 µL) and 2 mL of MeOH and allowed to stir for 30 min. After evaporation of the solvent, the crude was purified directly by chromatography on silica gel (chloroform/Methanol, 95:5), yielding 58% (32 mg), dr = 80/20.

**1-(2-(1,2-dihydroxyethyl)phenyl)-2-phenylethane-1,2-diol: (10)**

Yield: 32 mg (58%). Purification by flash chromatography on silica gel (DCM/MeOH, 95:5), obtained as white wax. Mixture of diastereomers (dr = 80/20). ^1^H NMR (600 MHz, MeOD-d_4_): δ 7.25–7.10 (m, 9H), 6.96 (td, *J* = 7.6, 1.4 Hz, 1H, major diastereomer), 6.50 (d, *J* = 7.6 Hz, 1H, minor diastereomer), 6.35 (d, *J* = 7.7 Hz, 1H, major diastereomer), 5.25 (d, *J* = 2.4 Hz, 1H, minor diastereomer), 5.13 (s, 1H, minor diastereomer), 5.09 (d, *J* = 3.6 Hz, 1H, major diastereomer), 5.05 (d, *J* = 3.6 Hz, 1H, major diastereomer), 4.85 (d, *J* = 1.5 Hz, 1H, minor diastereomer), 4.25 (s, 1H, minor diastereomer), 4.21 (d, *J* = 2.5 Hz, 1H, major diastereomer). ^13^C NMR (150 MHz, MeOD-d_4_) δ 140.7_major_, 140.6_minor_, 136.0_minor_, 133.9_major_, 132.7_minor_, 132.4_major_, 129.4_major_, 129.2_minor_, 127.6_minor_, 127.5_major_, 127.3_minor_, 127.0_major_, 127.0_minor_, 126.8, 126.8, 126.7, 126.7, 126.0_major_, 125.7_minor_, 93.3_major_, 93.0_minor_, 80.3_minor_, 76.4_major_, 76.3_major_, 68.3_mjor_. HRMS (MALDI-FT ICR): *m*/*z* calcd. for [C_16_H_18_NaO_4_]^+^: 297.1098; found: 297.1109.

## 4. Conclusions

We have described the vinylogous aldol reaction of isochromane-3,4-diones with both aromatic and aliphatic aldehydes. The reaction is facilitated by deprotonation at the γ-position of α-ketoester functionality within the isochromane-3,4-dione heterocyclic ring, allowing the in situ generation of dienol species that act as nucleophiles toward a wide range of aromatic and aliphatic aldehydes. Isochromane-3,4-diones bearing either electron-withdrawing or electron-donating substituents also afforded the corresponding vinylogous aldol products in a good yield. In most cases, the process was driven by the low solubility of the products in the reaction medium, resulting in a very high diastereoselectivity. The low solubility of the final products in common organic solvents limited follow-up chemistry, even if a reductive ring opening was achieved, leading to a novel tetrol derivative displaying a pinacol motif.

## Figures and Tables

**Table 1:** Reaction condition optimization.

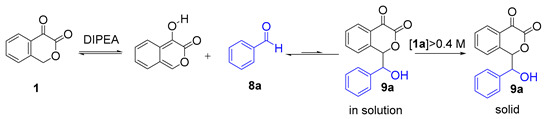							
Entry	Dione (Equiv.)	Benzaldehyde (Equiv.)	Base (Equiv.)	Solvent	Concentration Molarity	Time (h)	Yield (%)
**1**	1	1.3	DIPEA (2.0)	DCM	0.4	72	66 ^a^
**2**	1	1.3	DIPEA (2.0)	DCM	0.4	72	87 ^b^
**3**	1	1.3	DIPEA (2.0)	DCM	0.4	48	55 ^a^
**4**	2.3	1	DIPEA (1.0)	DCM	0.2	48	--
**5**	2.3	1	DIPEA (2.0)	DCM	0.2	5	20
**6**	1	1.3	DIPEA (2.0)	DCM	0.6	72	70
**7**	1	1.3	DIPEA (2.0)	CHCl_3_	0.4	72	65
**8**	1	1.3	NaH (1.5)	DMSO	0.2	24	--
**9**	1	1.3	K_2_CO_3_ (1.5)	ACN	0.2	24	--
**10**	2	1	Li_2_CO_3_ (1.2)	ACN	0.2	24	--

^a^ Isolated as solid from reaction mixture. ^b^ Overall yield from solid isolation and column chromatography of mother solution.

**Table 2:** Analysis of the scope.

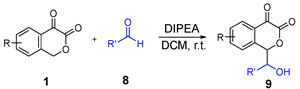
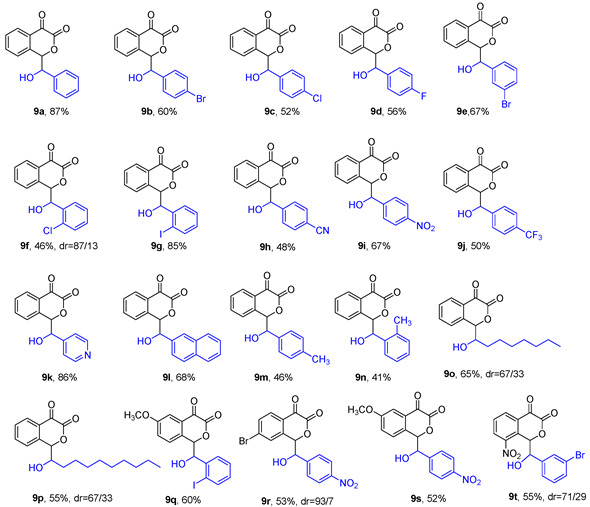

## Data Availability

The data that support the findings of this study are available in the Supplementary Material of this article. CCDC 2529279 contains the supplementary crystallographic data for compound **9a** of this paper. These data can be obtained free of charge from The Cambridge Crystallographic Data Centre. See Supporting Information for further details. Available online: http://www.ccdc.cam.ac.uk/structures (accessed on 10 April 2026).

## Supplementary Materials

The following supporting information can be downloaded at https://www.mdpi.com/article/10.3390/molecules31091508/s1: X-ray crystallography of **9a**. Copies of ^1^H and ^13^C NMR spectra of all the new compounds. The authors have cited additional references within the Supporting Information [28,29,30,31,32,33].
