# Supplementary material for: Isochromane-3,4-diones in Direct Vinylogous Aldol Reaction: Scope and Limitations
Source: Molecules. 2026 May 1;31(9):1508. doi: 10.3390/molecules31091508 (PMC13164694; doi:10.3390/molecules31091508)
Supplement: Supplementary file 1 [file molecules-31-01508-s001.zip › molecules-4299984-Supplementary.pdf]

# Supporting Information

## Isochromane-3,4-diones in vinylogous direct aldol reaction

Sanam Gull Arshad, Mohammed Sadeq Mousavi, Consiglia Tedesco and Antonio Massa*

[a] Dipartimento di Chimica e Biologia "A. Zambelli" Università degli Studi di Salerno, Via Giovanni Paolo II, 84084-Fisciano (SA) (Italy)
E-mail: amassa@unisa.it

## Table of Contents

1. General 2
2. X-ray Crystallography 3
3. Copies of NMR Spectra 6
4. References 30

## General

Unless otherwise noted, all chemicals, reagents and solvents for the performed reactions are commercially available. 3-Isochromanone was purchased from Fluorochem. Isochromane-3,4-dione and substituted isochromane-3,4-diones were prepared according to literature procedures [1-3]. All the reactions were monitored by thin layer chromatography (TLC) on precoated silica gel plates (0.25 mm) and visualized by fluorescence quenching at 254 nm. Flash chromatography was carried out using neutral activated alumina. (Merck, Darmstadt, Germany). Yields are given for isolated products showing one spot on a TLC plate. The NMR spectra were recorded on Bruker DRX 600, 400, 300 and 250 MHz spectrometers (600 MHz, $^{1}$H, 150 MHz, $^{13}$C; 400 MHz, $^{1}$H, 100.6 MHz; $^{13}$C, 300 MHz, $^{1}$H, 75.5 MHz, $^{13}$C, 250 MHz, $^{1}$H, 62.5 MHz, $^{13}$C). Internal reference was set to the residual solvent signals ($\delta$H 7.26 ppm, $\delta$C 77.16 ppm for CDCl3, $\delta$H 2.50 ppm, $\delta$C 39.10 ppm for DMSO-d6). The $^{13}$C NMR spectra were recorded under broad-band proton decoupling. $^{1}$HNMR, $^{13}$CNMR and HRMS data are provided for all newly synthesized compounds. Copies of $^{1}$H and $^{13}$C NMR spectra for these compounds are included in the supplementary materials. The following abbreviations are used to indicate the multiplicity in NMR spectra: s singlet, d-doublet, t-triplet, q-quartet, dd-doublet of doublets, m-multiplet, br s-broad signal. High resolution mass spectra (HRMS) were acquired using a Bruker SolariX XR Fourier transform ion cyclotron resonance mass spectrometer (Bruker Daltonik GmbH, Bremen, Germany) equipped with a 7T refrigerated actively shielded superconducting magnet. For ionization of the samples electrospray ionization (ESI) or MALDI was applied.

**Single crystal X-ray diffraction**

Single crystals of **9a** were obtained by slow evaporation of (5 mg) in a Hexane/MeOH (1.5 mL, 2:1 v/v).

A suitable single crystal of **9a** was selected and mounted in a cryoloop with Paratone oil and data collected under a cold nitrogen flow (Oxford Cryostream 800) at 100 K by means of a Bruker D8 QUEST diffractometer equipped with a PHOTON detector using Cu-Ka radiation ($\lambda$= 1.54178 Å). Data indexing was performed using APEX3 software.[4] Data integration and reduction were performed using SAINT.[5] Absorption correction was performed by multi-scan method in SADABS.[6] The structure was solved using SHELXS[7] and refined by means of full matrix least-squares based on $F^5$ using the program SHELXL.[8] OLEX2 was used as GUI.[9]

There are two crystallographically independent molecules (named with suffix A and B). Moreover, one aromatic ring in molecule B is affected by disorder with three atoms in two possible locations (at least). Thus, non-hydrogen atoms were refined anisotropically, hydrogen atoms were positioned geometrically and included in structure factors calculations with the exception of the H1 hydrogen atom, covalently bound to hydroxyl oxygen atom O1 and hydrogen-bonded to O3 carbonyl oxygen atom, whose coordinates were isotropically refined.

ORTEP diagram was drawn using OLEX2 (Figure S1).[9] Relevant crystallographic data and refinement details are reported in Table S1.

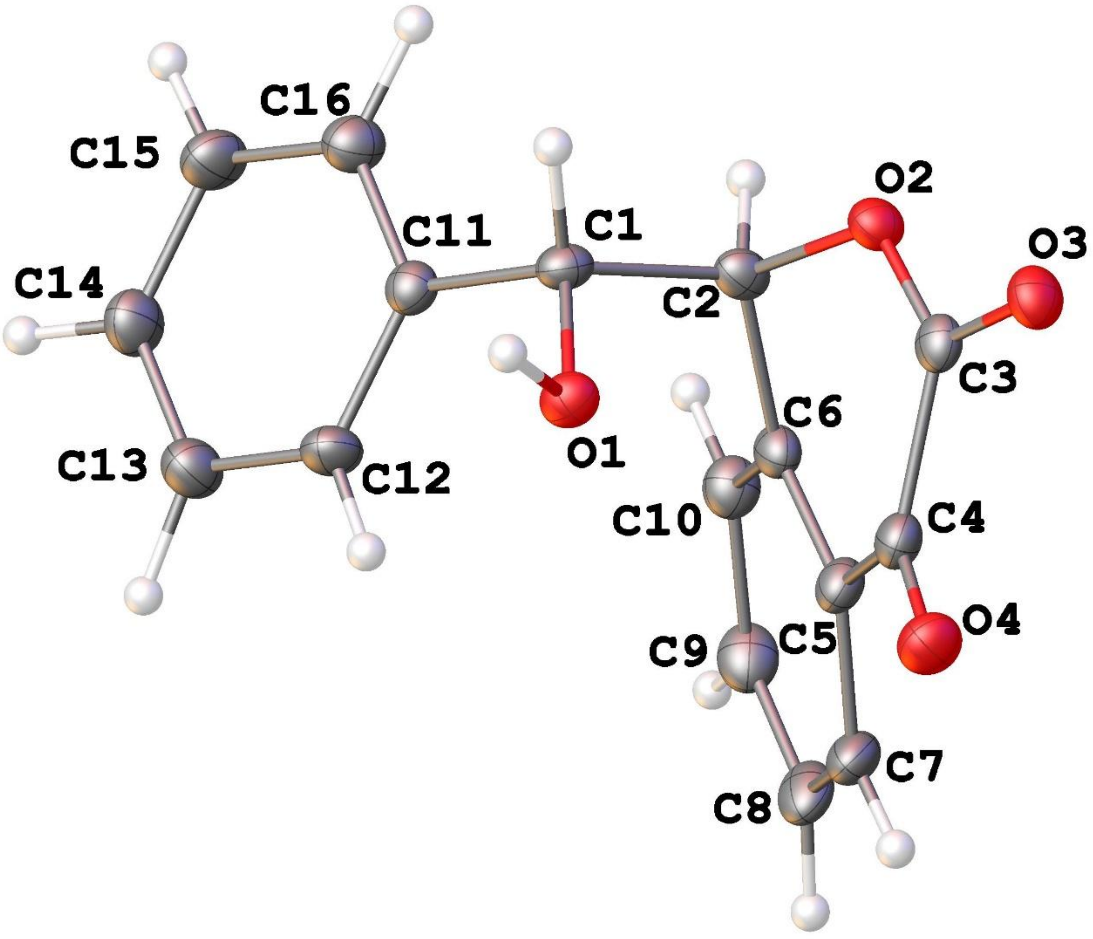


**Figure S1.** ORTEP drawing of compound **9a**. Ellipsoids are shown at 50 % probability level (CCDC code: 2529279).

**Table S1. Relevant crystallographic data and refinement details for compound 9a.**

| | |
|---|---|
| Compound | 9a |
| CCDC code | 2529279 |
| Empirical formula | $C_{16}H_{12}O_4$ |
| Formula weight | 268.26 |
| Temperature (K) | 100 |
| Crystal system | orthorhombic |
| Space group | $Pna2_1$ |
| $a$(Å) | 16.0431(6) |
| b (Å) | 14.1179(5) |
| $c$ (Å) | 5.4353(2) |
| $V$ (Å$^3$) | 1231.07(8) |
| $Z$ | 4 |
| $\rho_{calc}$ (g/cm$^3$) | 1.447 |
| μ (mm$^{-1}$) | 0.865 |
| F(000) | 560.0 |
| Radiation | CuKα (λ = 1.54178 Å) |
| 2Θ range for data collection/° | 8.342 to 142.934 |
| Index ranges | $-18 \leq h \leq 19$, $-17 \leq k \leq 17$, $-6 \leq l \leq 4$ |
| Reflections collected | 5401 |
| Independent reflections | 1881 [$R_{int}$ = 0.0431, $R_{sigma}$ = 0.0484] |
| Data/restraints/parameters | 1881/1/186 |
| Goodness-of-fit on $F^2$ | 1.189 |
| Final R indexes [I>=2σ (I)] | $R_1$ = 0.0575, $wR_2$ = 0.1351 |
| Final R indexes [all data] | $R_1$ = 0.0632, $wR_2$ = 0.1452 |
| Largest diff. peak/hole (e Å$^{-3}$) | 0.64/-0.53 |
| Flack parameter | 0.11(13) |

## 1-(hydroxy(phenyl)methyl)isochromane-3,4-dione (9a)

$^1$H NMR (400 MHz, DMSO-d6)

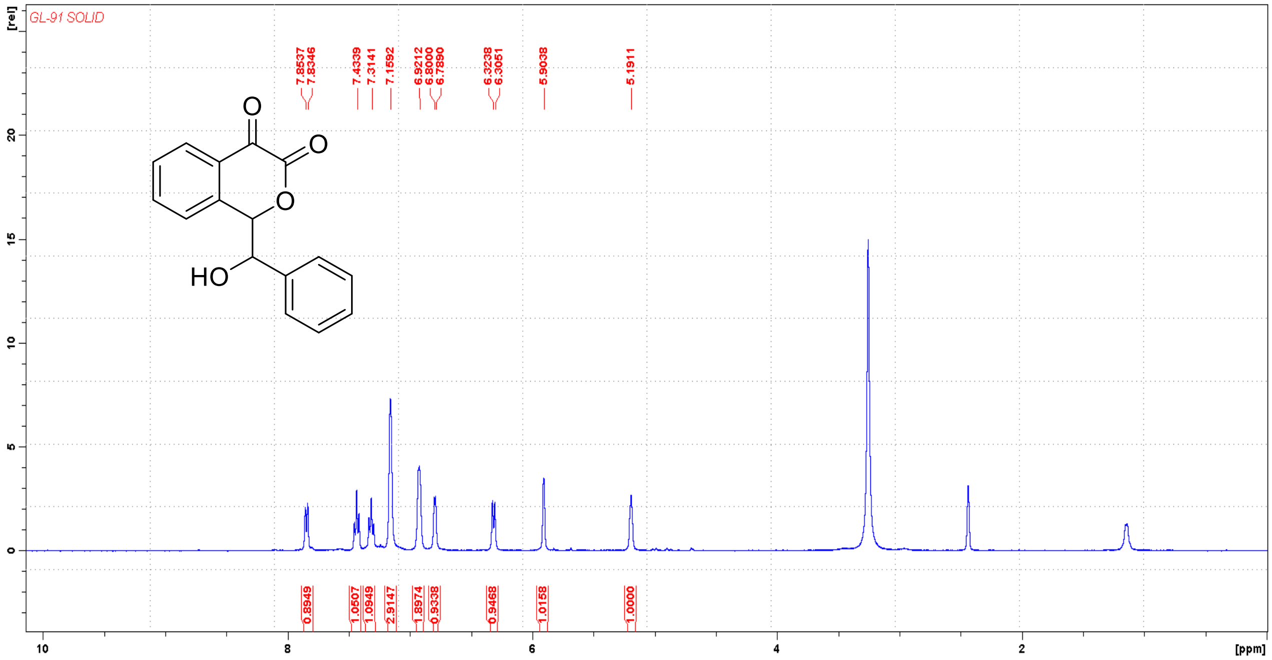


$^{13}$C{$^1$H} NMR (100 MHz, DMSO-d6)

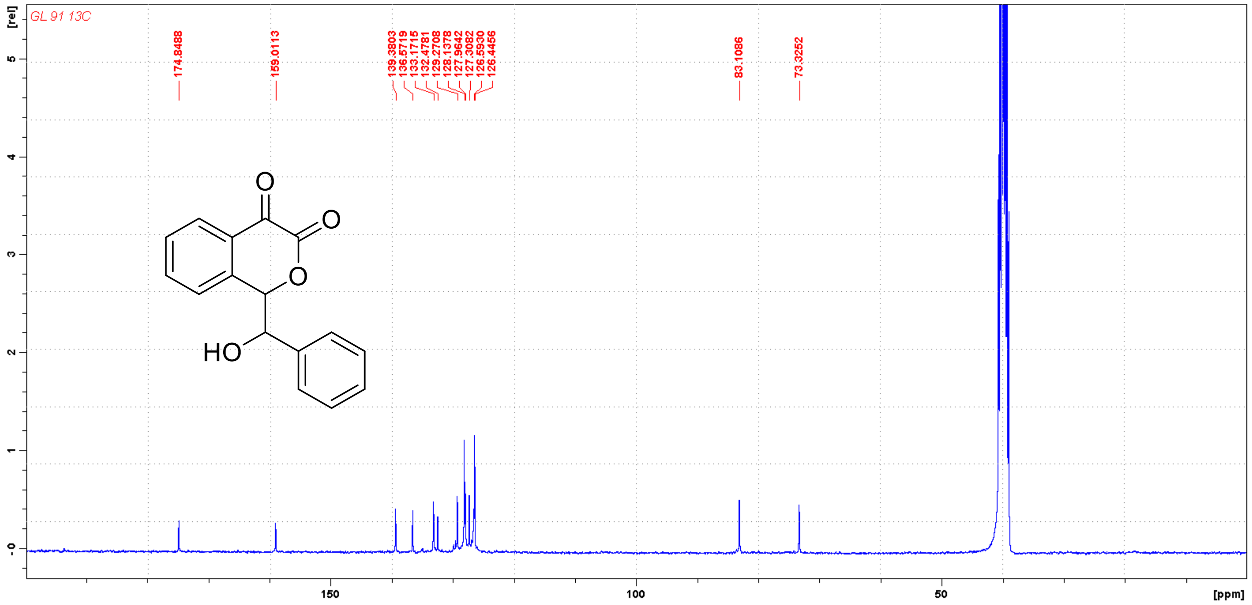

## HRMS for 9a

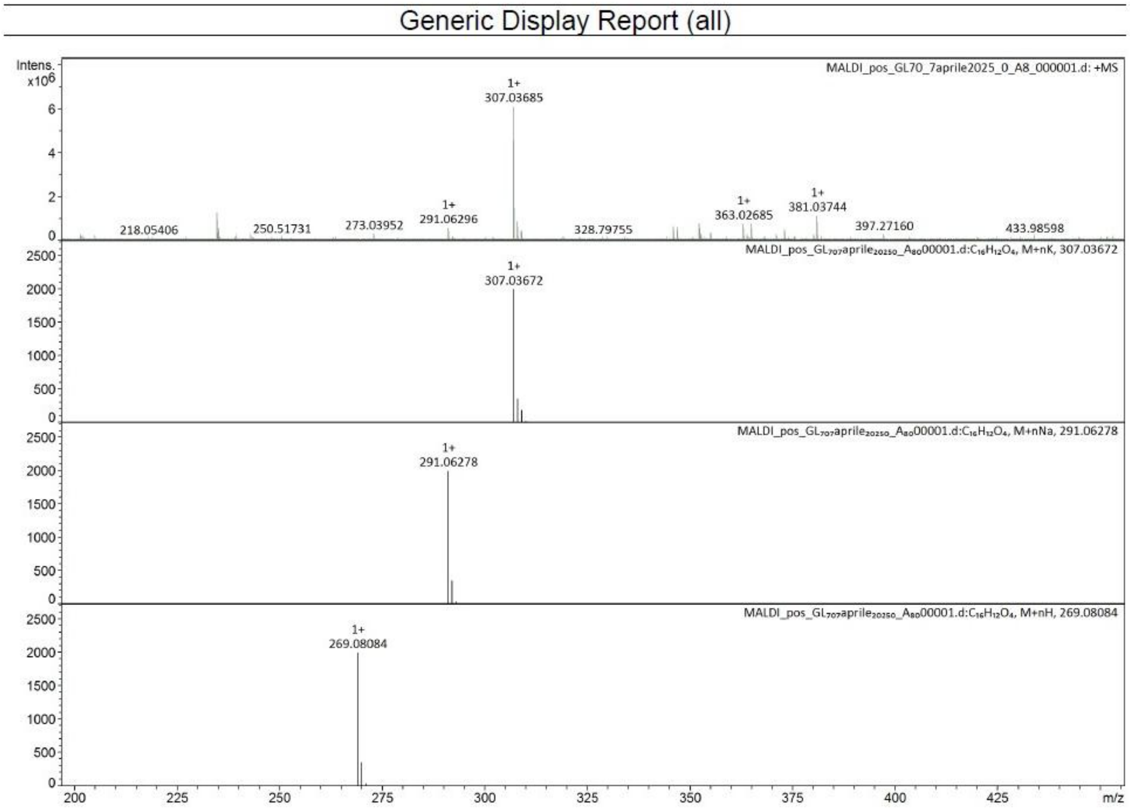
Generic Display Report (all)
MALDI_pos_GL70_7aprile2025_0_A8_000001.d: +MS
307.03685
291.06296
363.02685
381.03744
M+nK, 307.03672
M+nNa, 291.06278
M+nH, 269.08084
m/z

## 1-((4-bromophenyl)( hydroxy) methyl) isochromanone-3,4 dione (9b)

$^{1}H$ NMR (400 MHz, DMSO-d6)

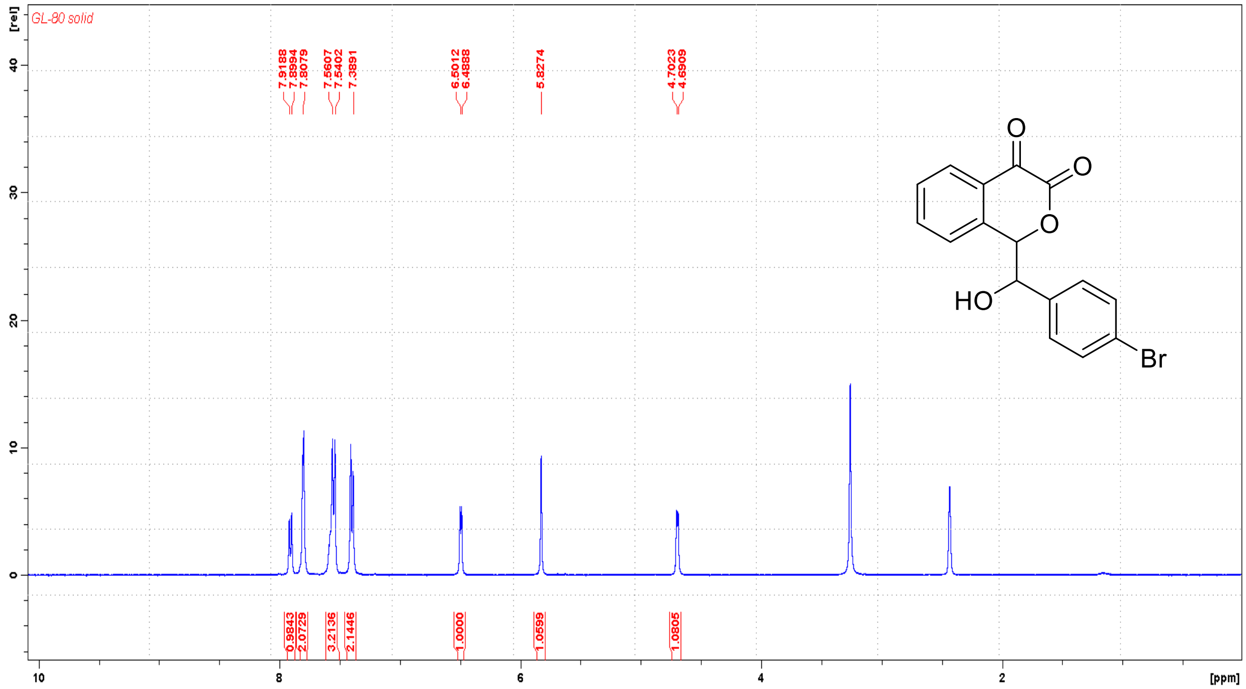


$^{13}C\{^{1}H\}$ NMR (150 MHz, DMSO-d6)

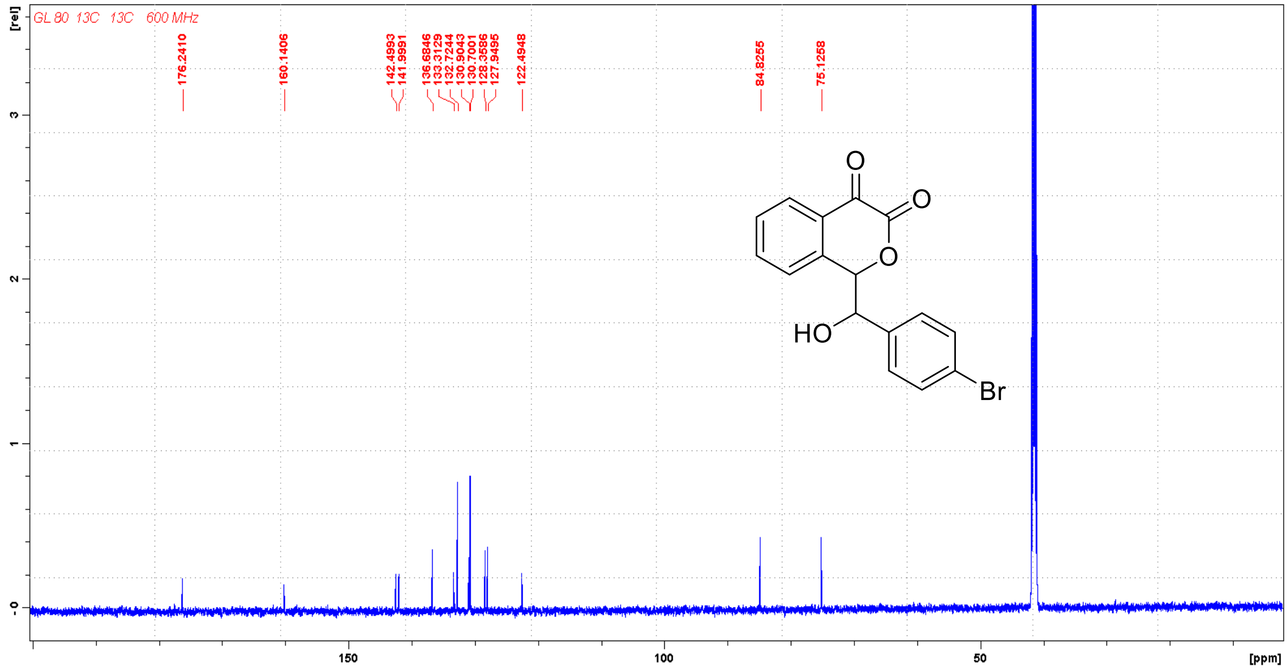

**1-((4-chlorophenyl)(hydroxy)methyl)isochromane-3,4-dione (9c)**

$^{1}$H NMR (300 MHz, DMSO-d6)

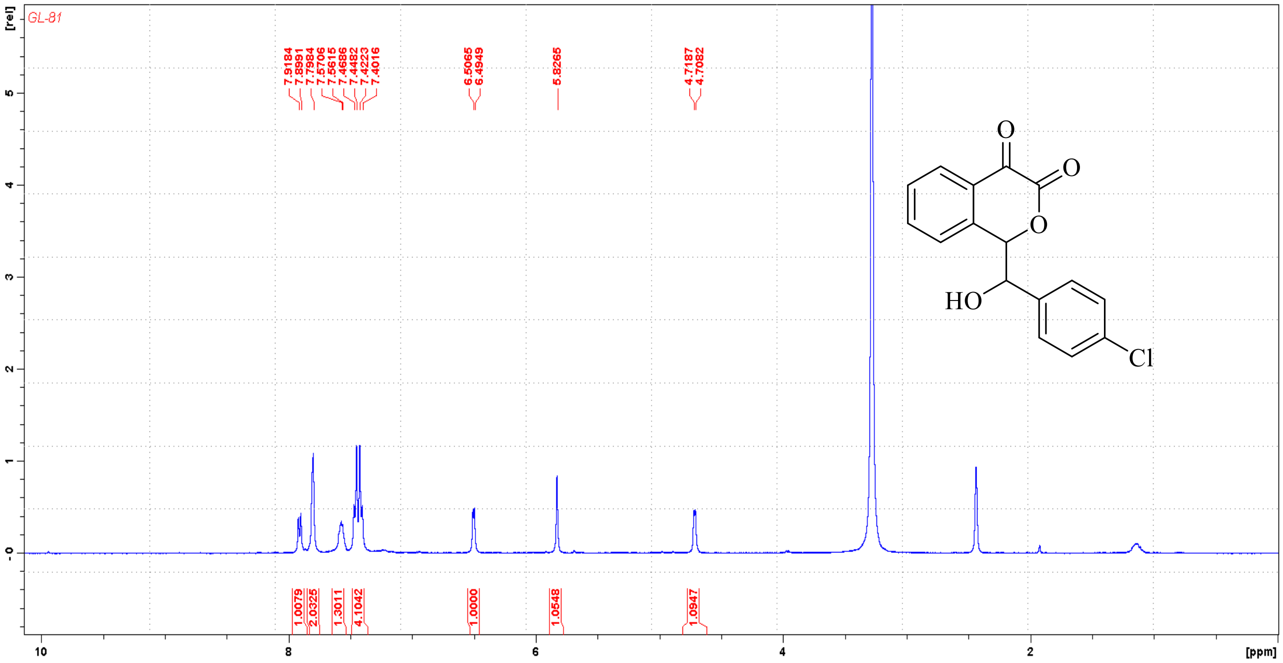


$^{13}$C{$^{1}$H} NMR (100 MHz, DMSO-d6)

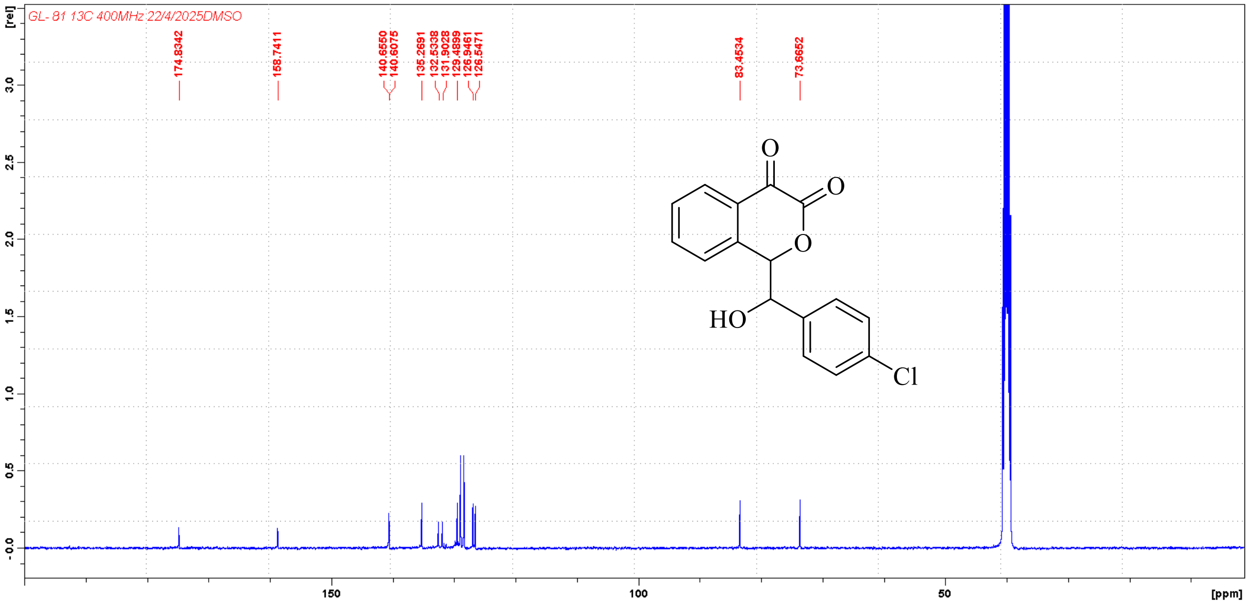

**1-((4-flluorophenyl) (hydroxy)methyl)isochromanone-3,4 dione (9d)**

$^{1}H$ NMR (300 MHz, DMSO-d6)

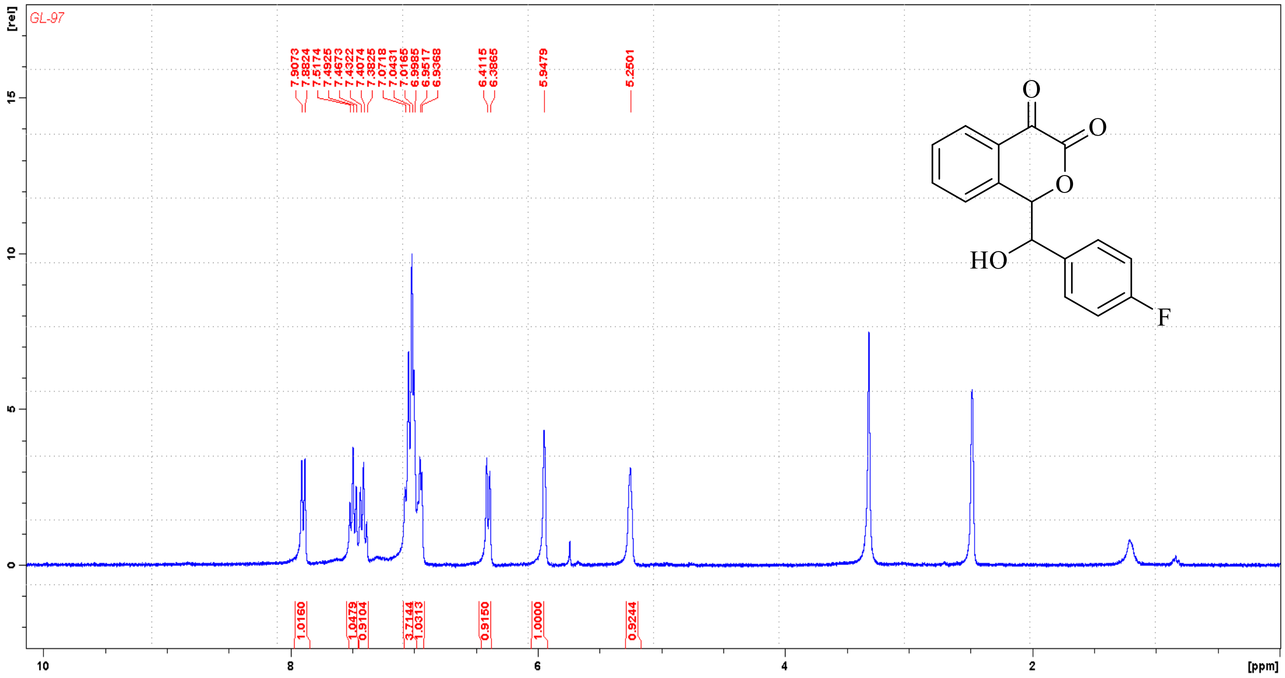

$^{13}C\{^{1}H\}$ NMR (150 MHz, DMSO-d6)

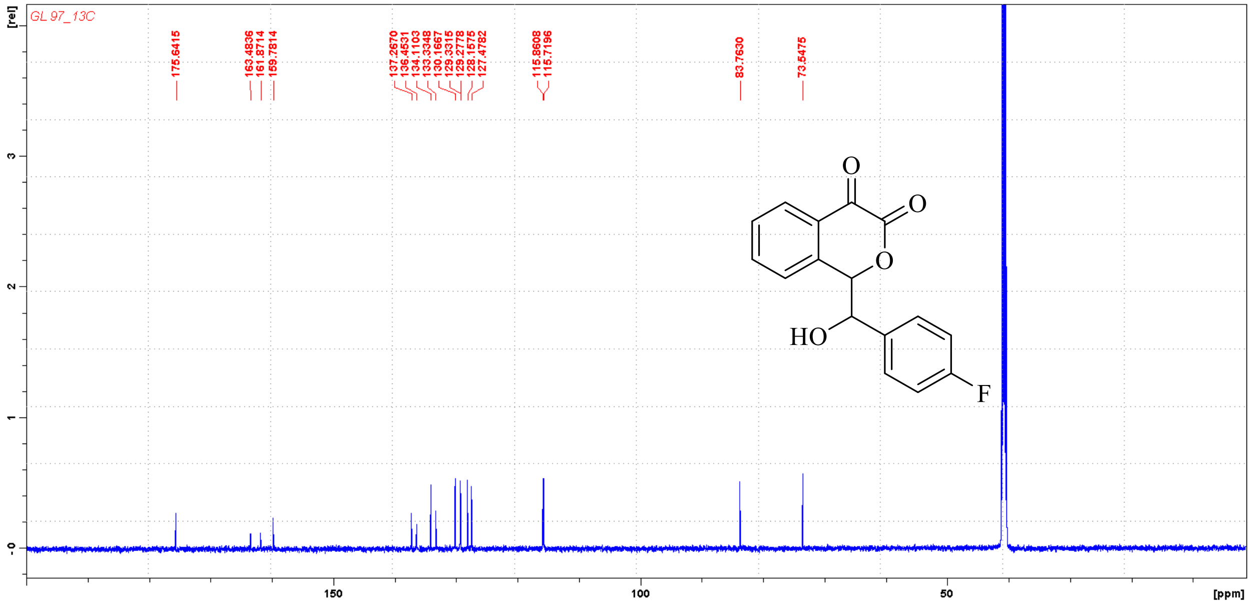

$^{19}F\{^{1}H\}$ NMR (376 MHz, DMSO-d6)

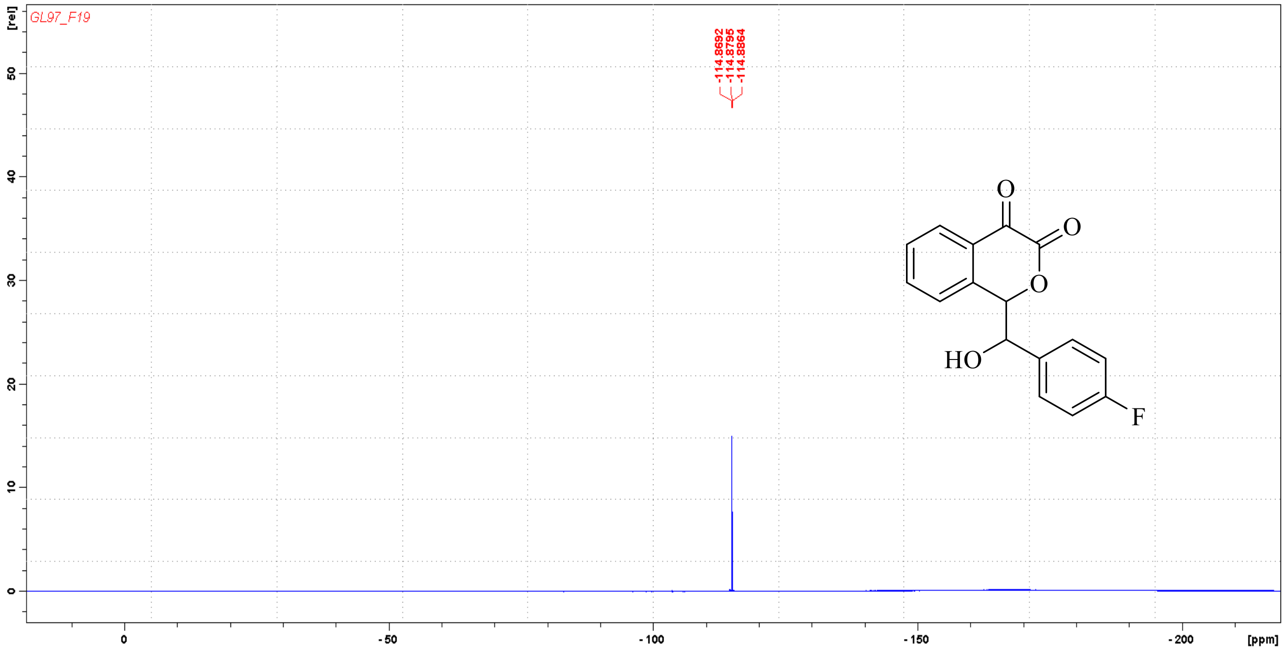

**1-((3-bromophenyl)( hydroxy)methyl)isochromanone-3,4 dione (9e)**

$^{1}H$ NMR (300 MHz, DMSO-d6)

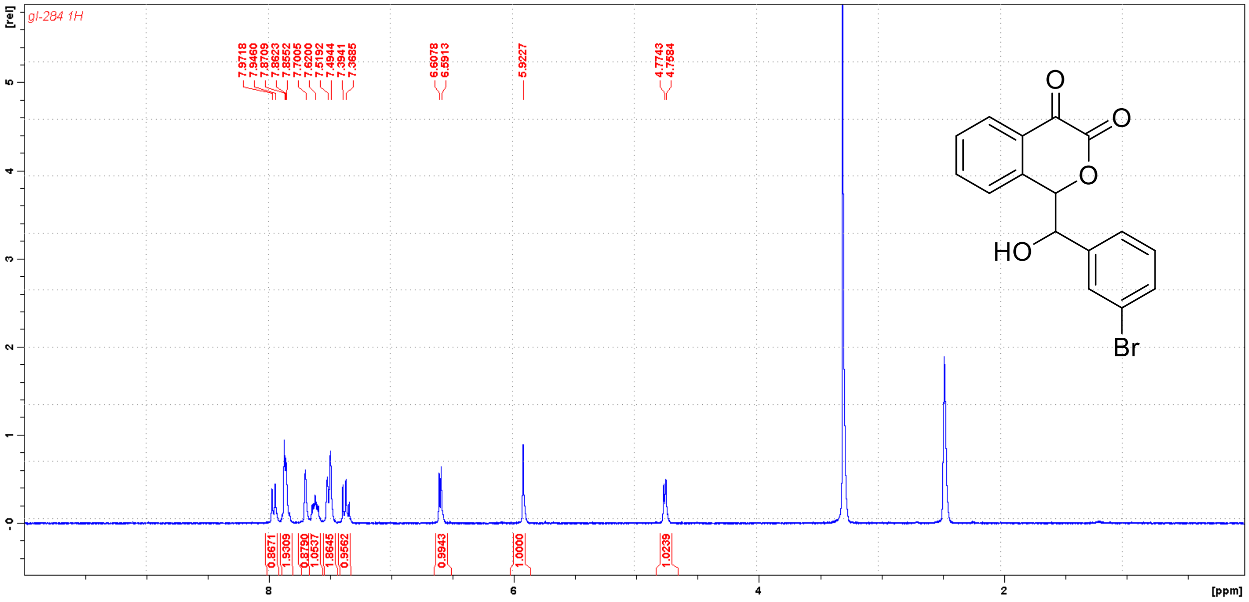


$^{13}C\{^{1}H\}$ NMR (100 MHz, DMSO-d6)

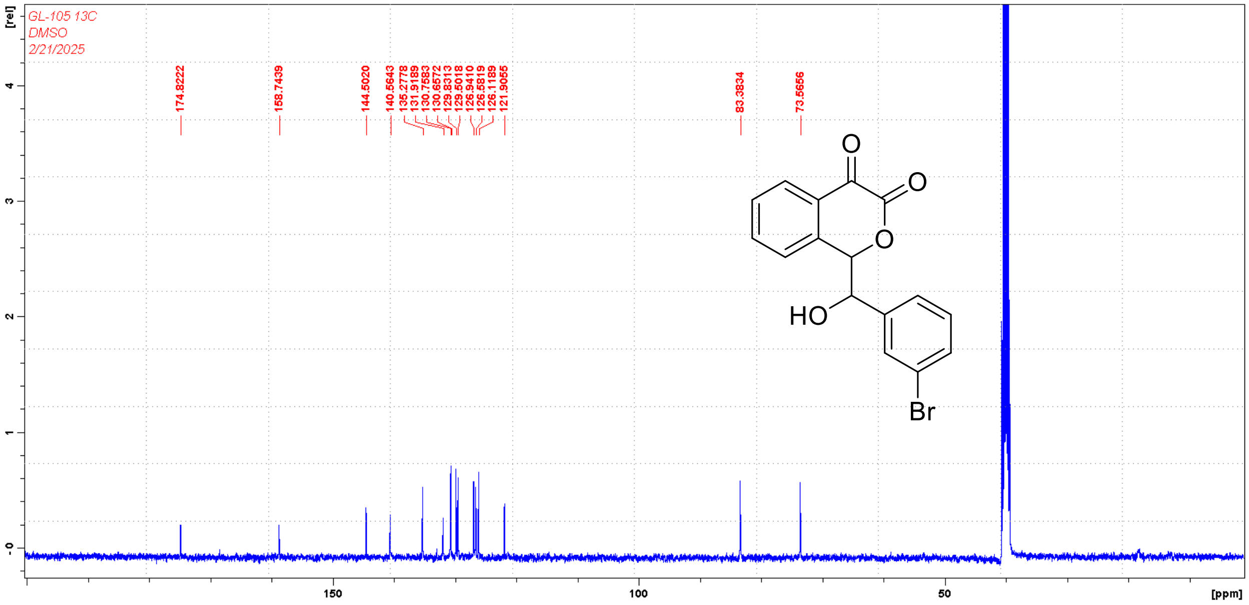

**1-((2-chlorophenyl)(hydroxy)methyl)isochromane-3,4-dione (9f)**

$^{1}$H NMR (400 MHz, DMSO-d6)

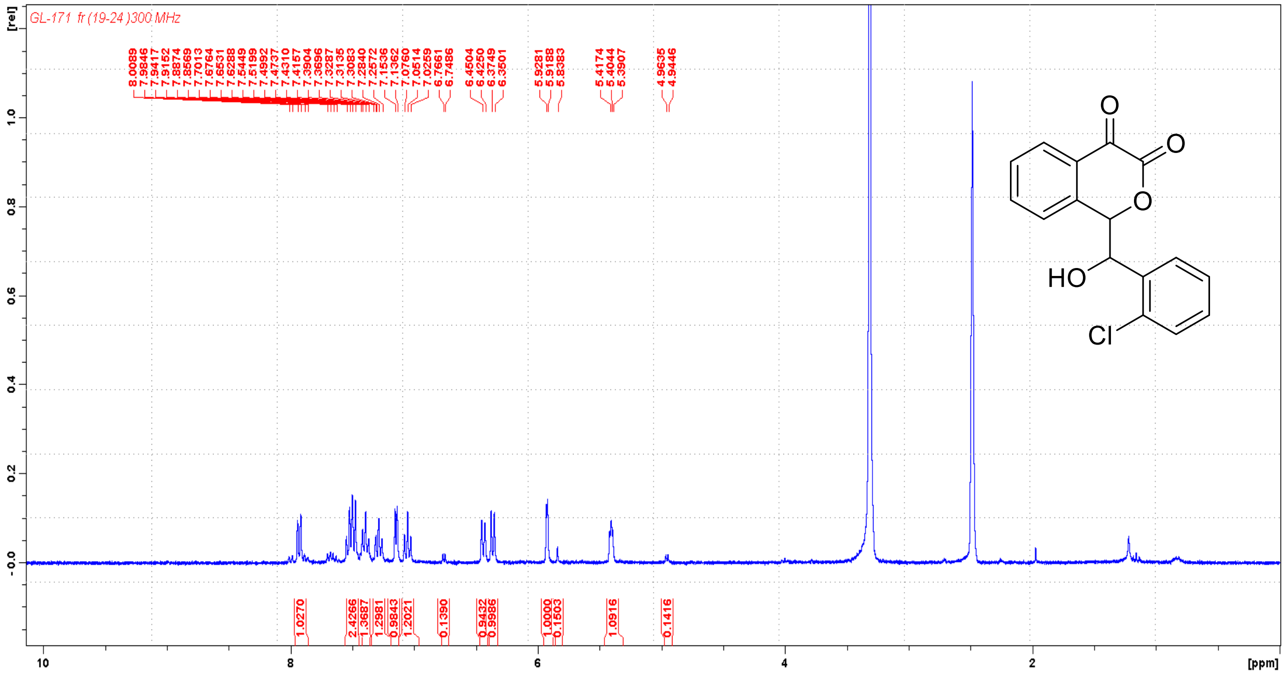

$^{13}$C{$^{1}$H} NMR (63 MHz, DMSO-d6)

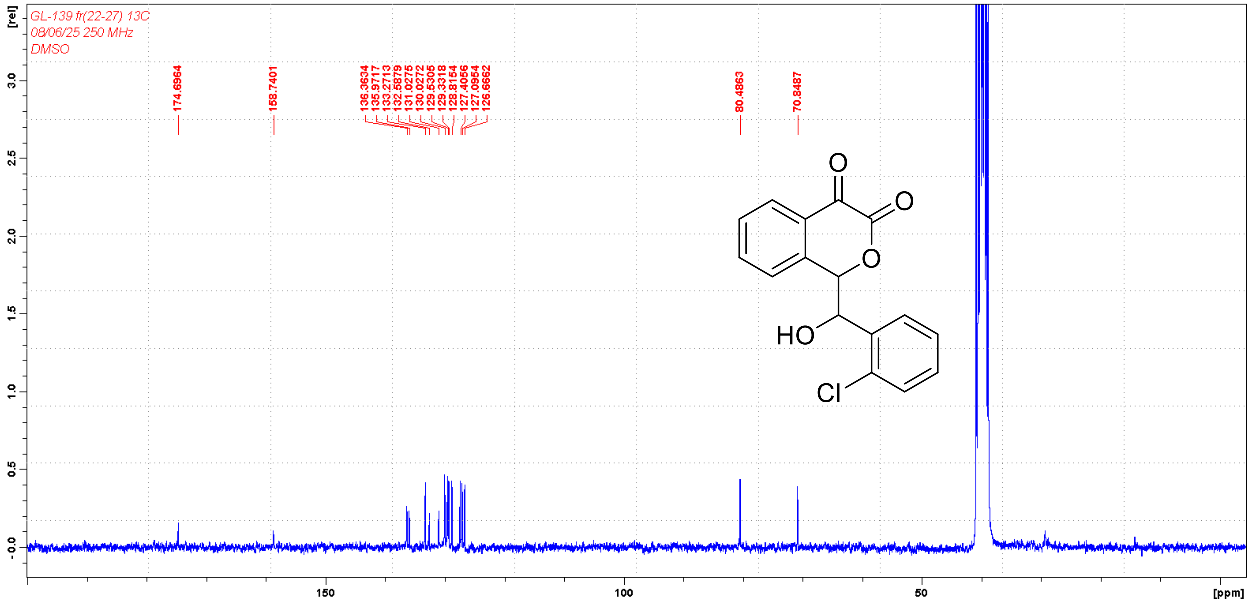

**1-(hydroxy(2-iodophenyl),ethyl)isochromanone-3,4-dione (9g)**

$^{1}H$ NMR (400 MHz, DMSO-d6)

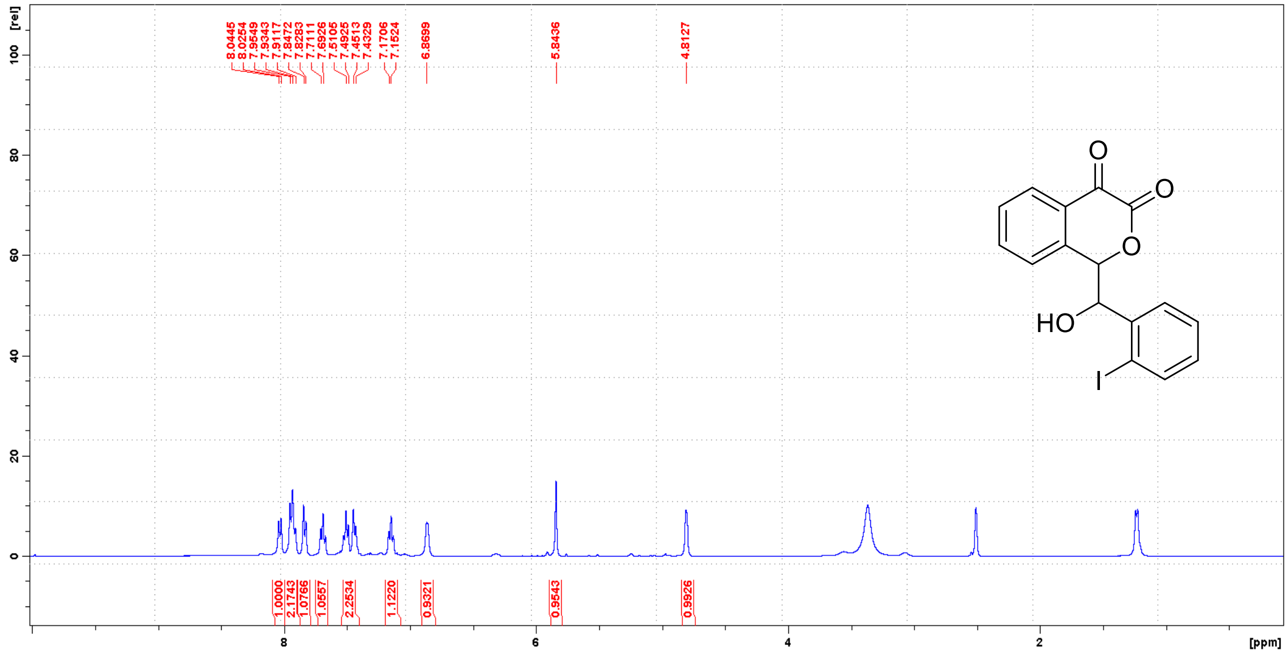

$^{13}C\{^{1}H\}$ NMR (100 MHz, DMSO-d6)

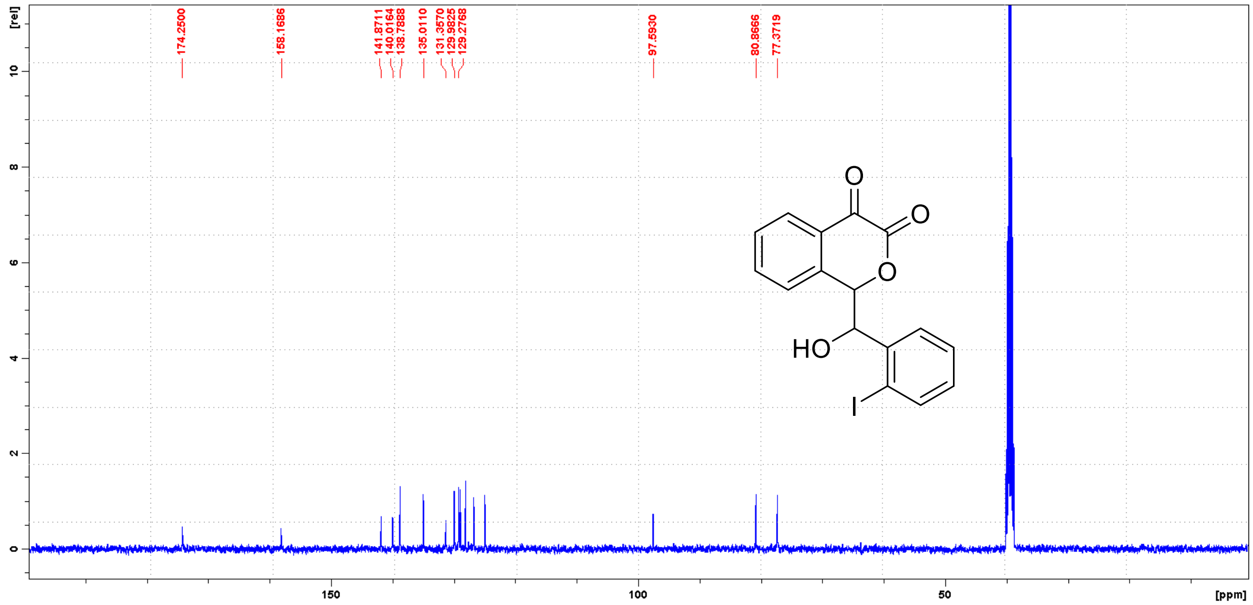

## 4-((3,4-dioxoisochroman-1-yl)(hydroxy)methyl) benzonitrile (9h)

$^1$H NMR (400 MHz, DMSO-d6)

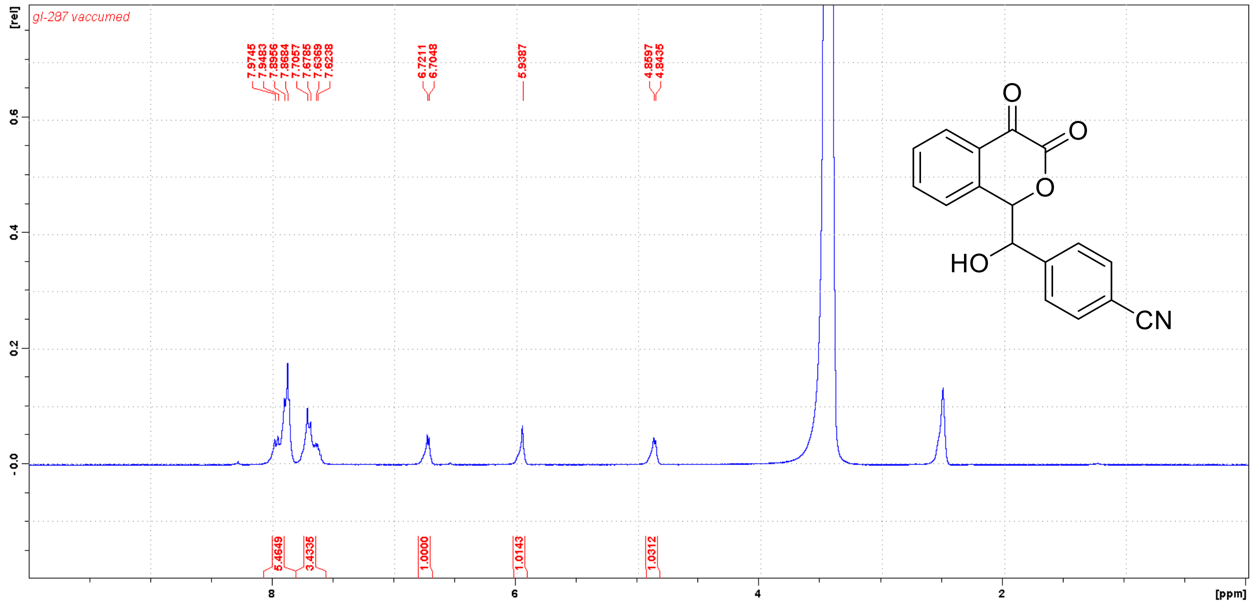


$^{13}$C{$^1$H} NMR (63 MHz, DMSO-d6)

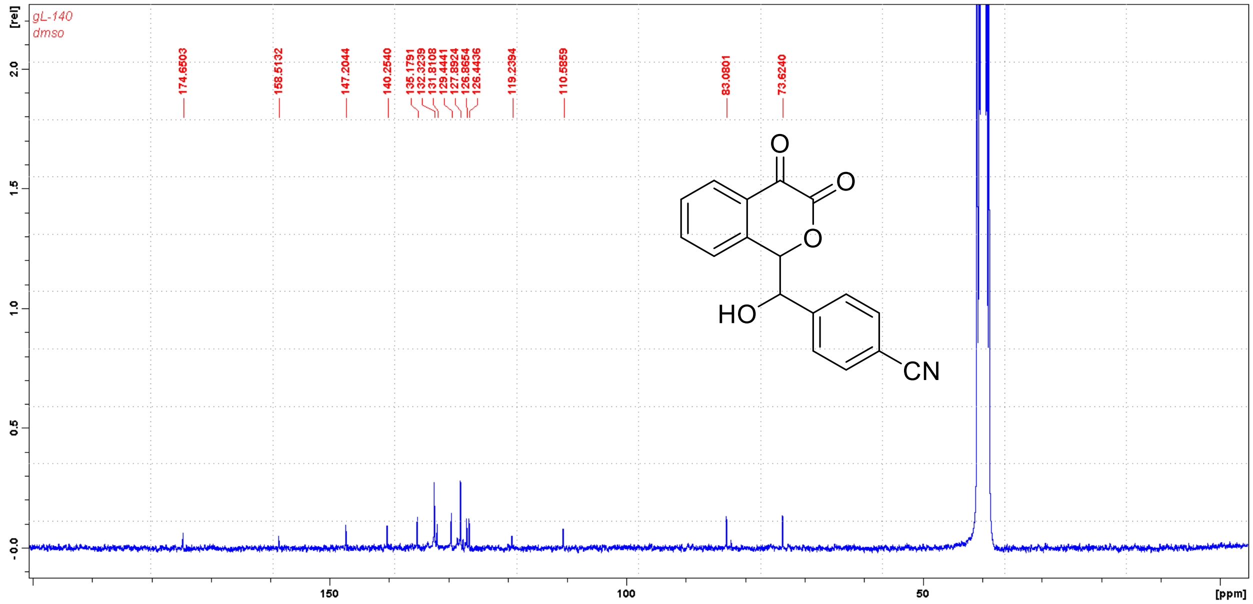

## 1-hydroxy(4-nitrophenyl) methyl) isochromanone-3,4 dione (9i)

$^{1}H$ NMR (400 MHz, DMSO-d6)

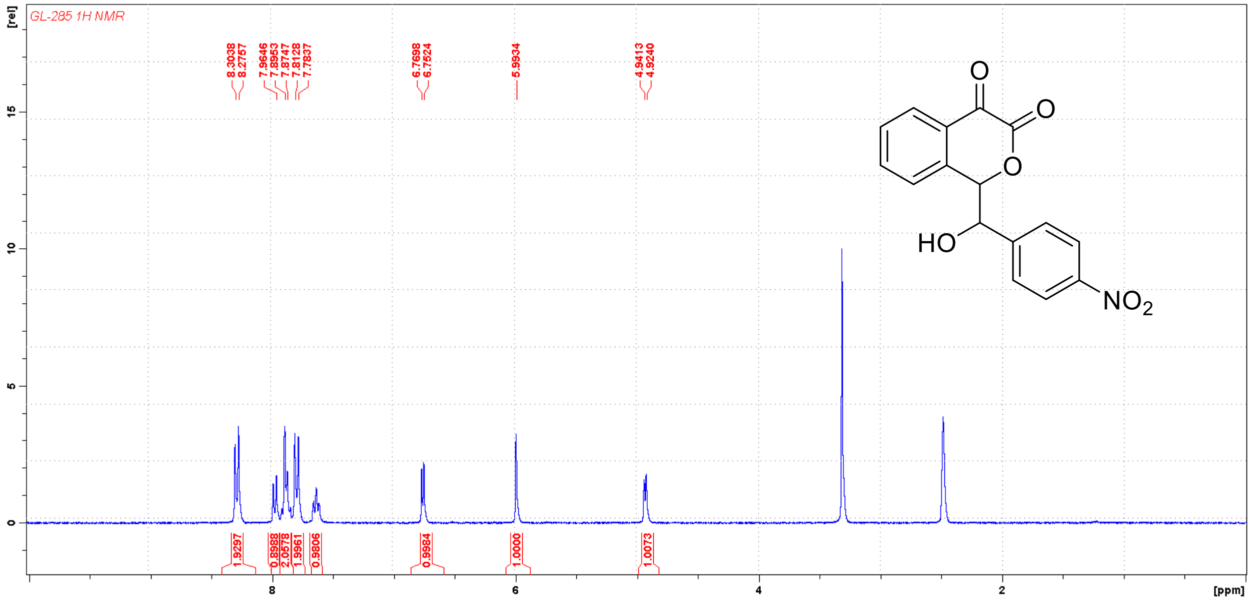


$^{13}C\{^{1}H\}$ NMR (75 MHz, DMSO-d6)

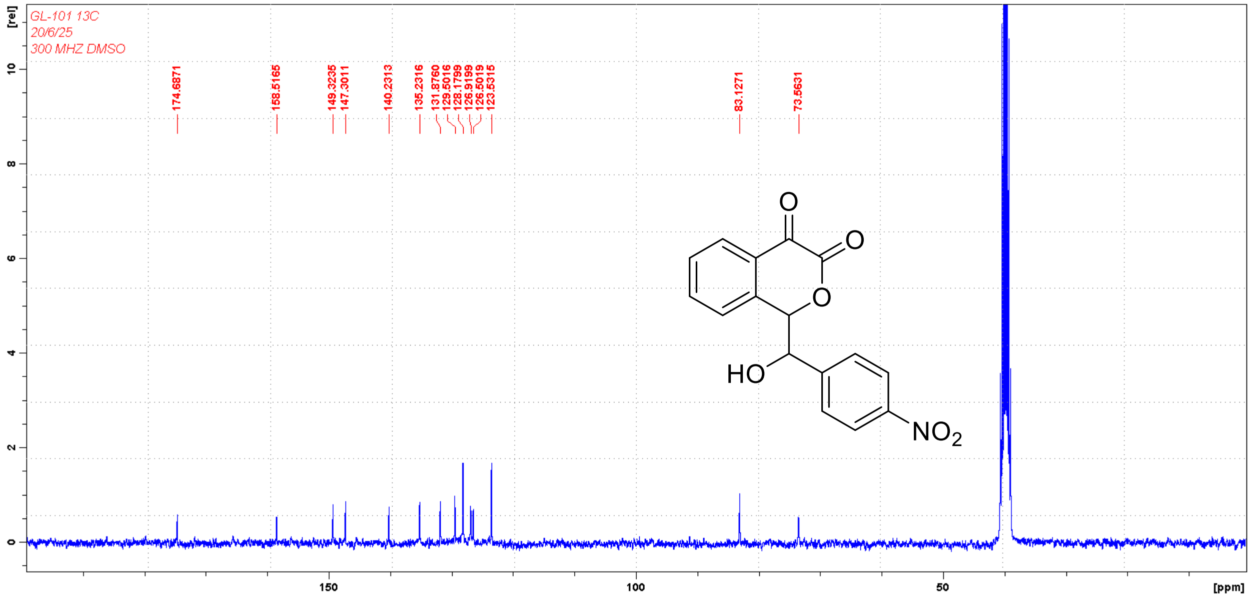

**1-hydroxy(2-trifluromethyl) phenyl)isochromanone-3,4 dione (9j)**

$^{1}$H NMR (300 MHz, DMSO-d6)

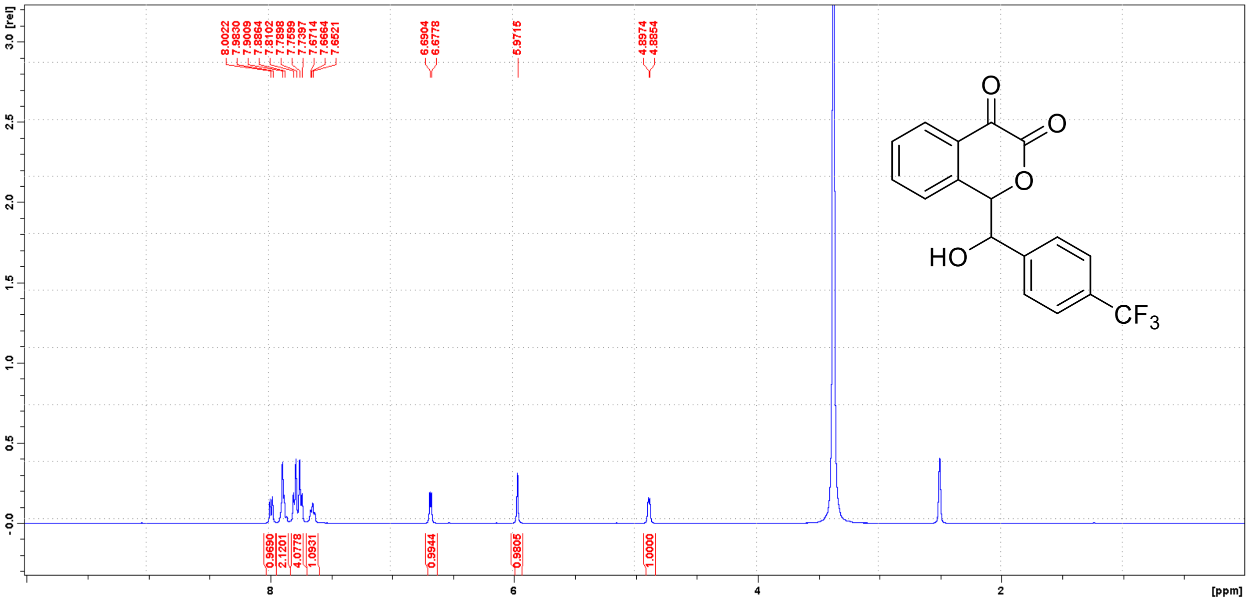

$^{13}$C{$^{1}$H} NMR (150 MHz, DMSO-d6)

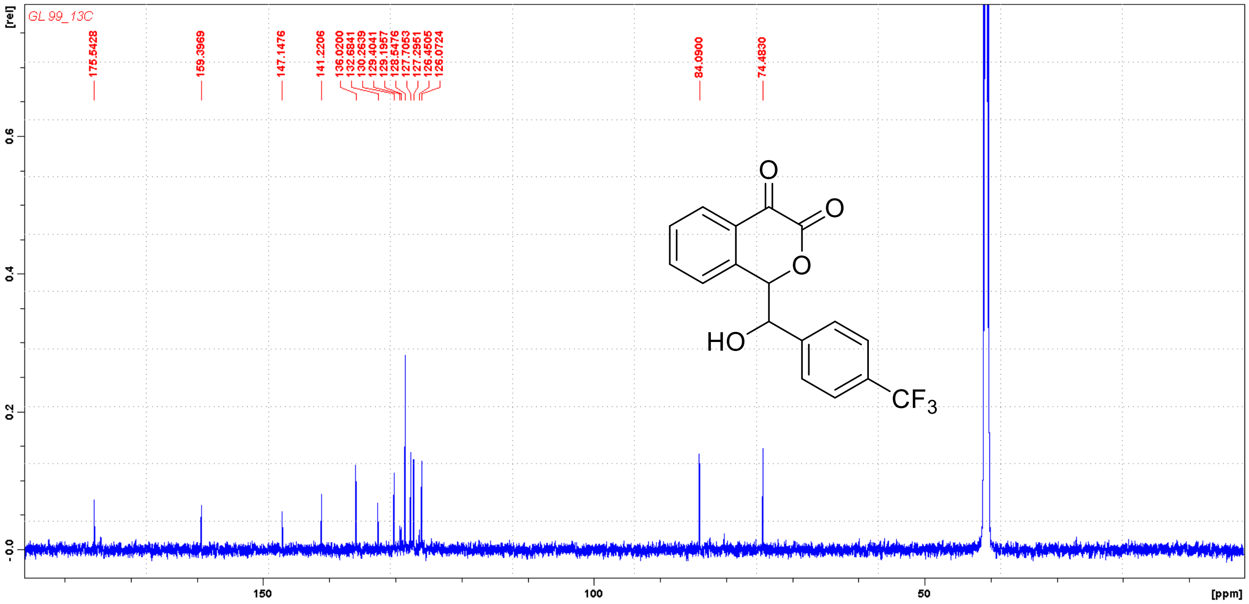

$^{19}F\{^1H\}$ NMR (376 MHz, DMSO-d6)

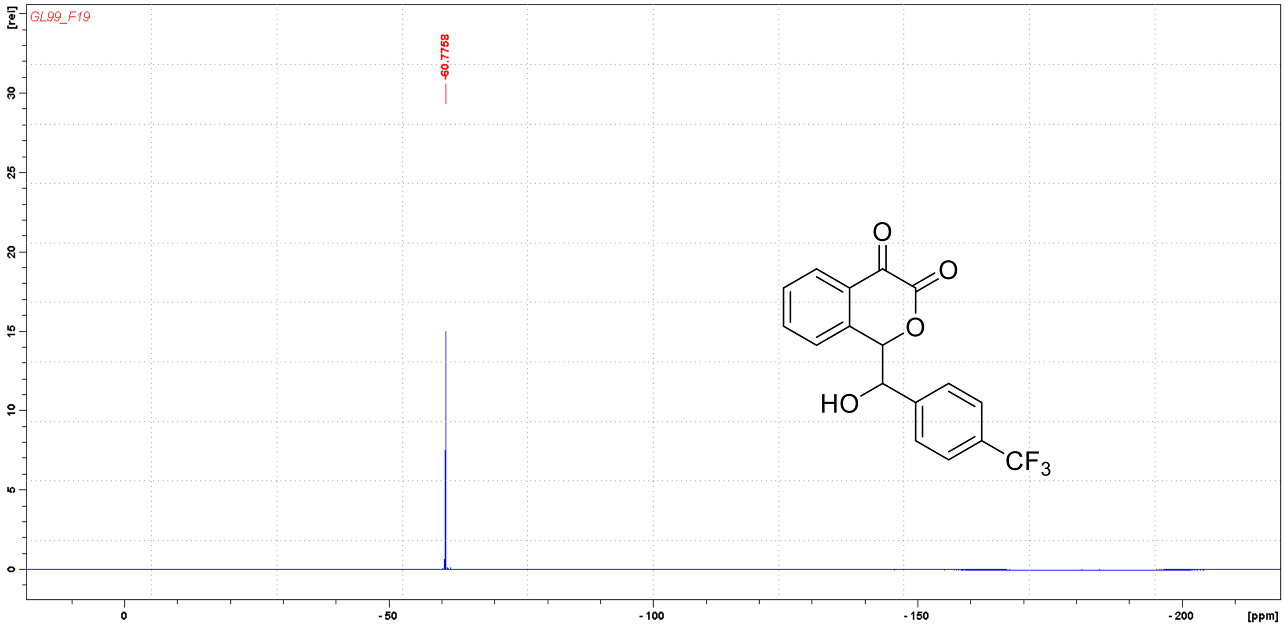

## 1-(hydroxy(pyridin-4-yl)methyl)isochromane-3,4-dione (9k)

$^{1}$H NMR (400 MHz, MeOD-d4/DMSO-d6)

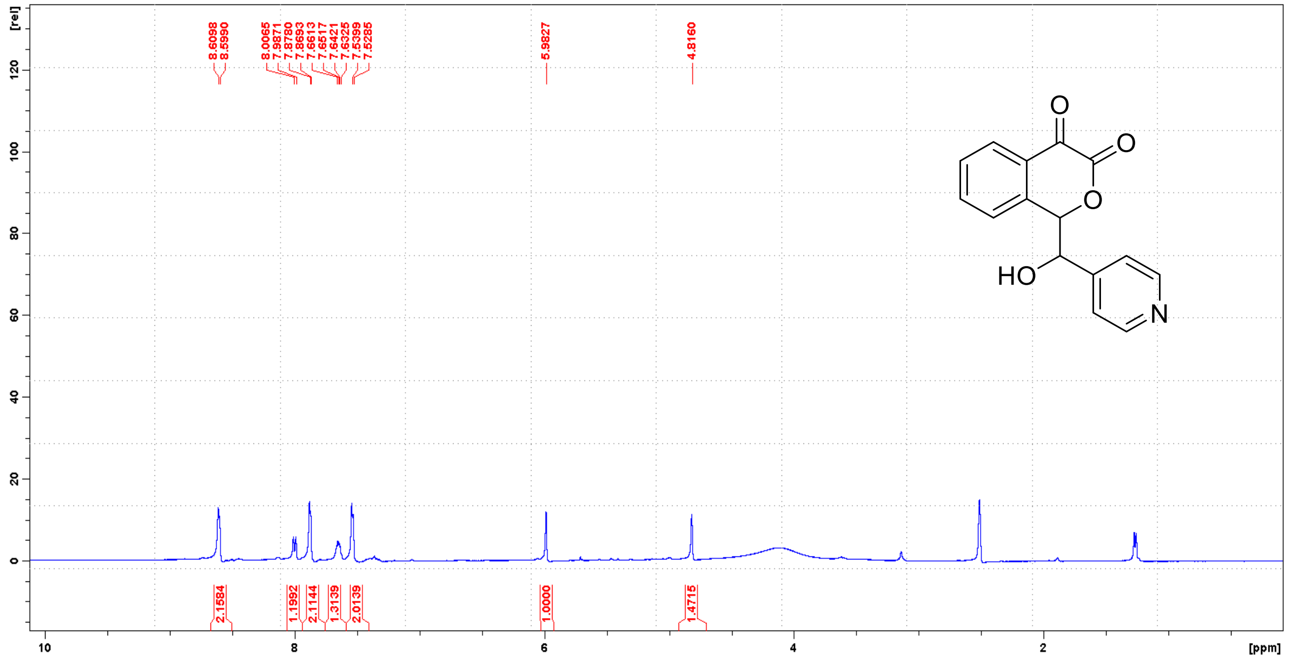

$^{13}$C{$^{1}$H} NMR (100 MHz, MeOD-d4/DMSO-d6)

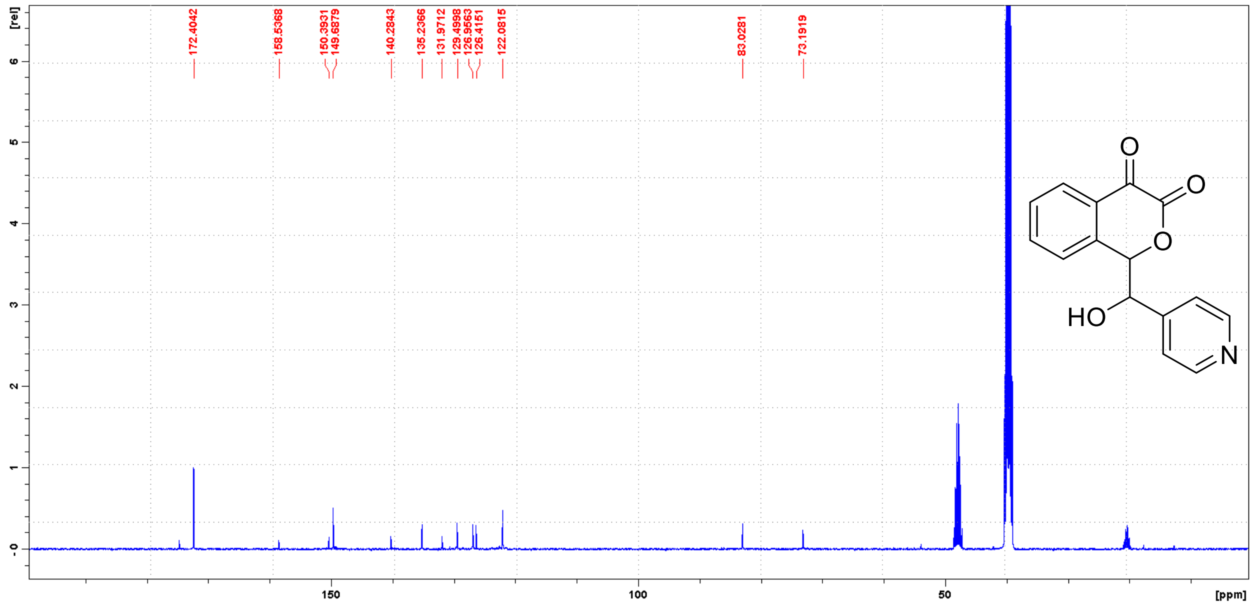

## 1-(hydroxy(naphthalen-2-yl)methyl)isochromane-3,4-dione (9l)

$^{1}H$ NMR (300 MHz, MeOD-d4)

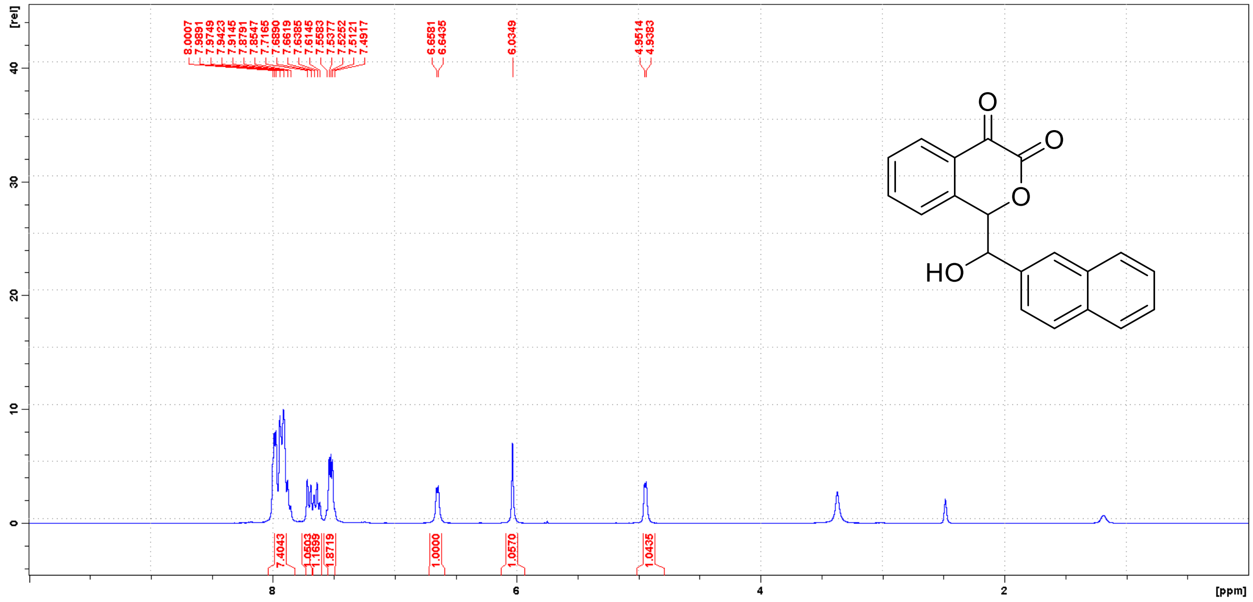

$^{13}C\{^{1}H\}$ NMR (100 MHz, MeOD-d4)

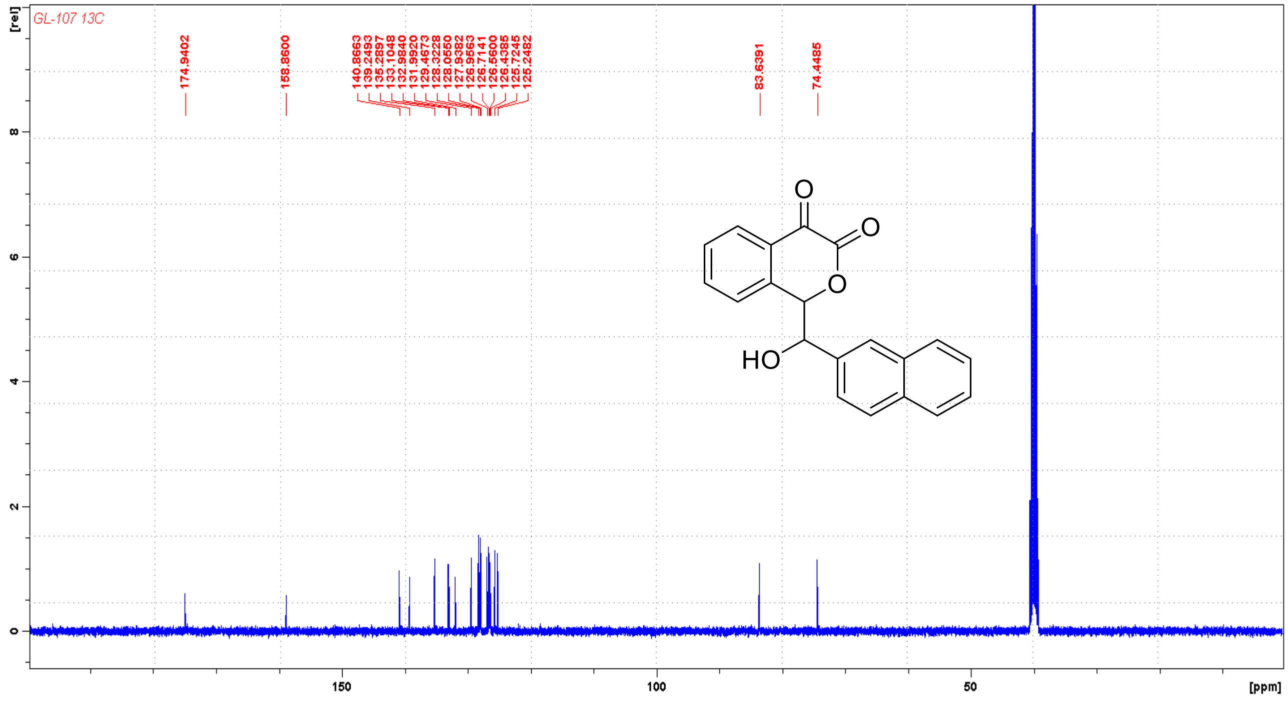

## 1-(hydroxy(p-tolyl) methyl) isochromanone-3,4 dione (9m)

$^1$H NMR (600 MHz, DMSO-d6)

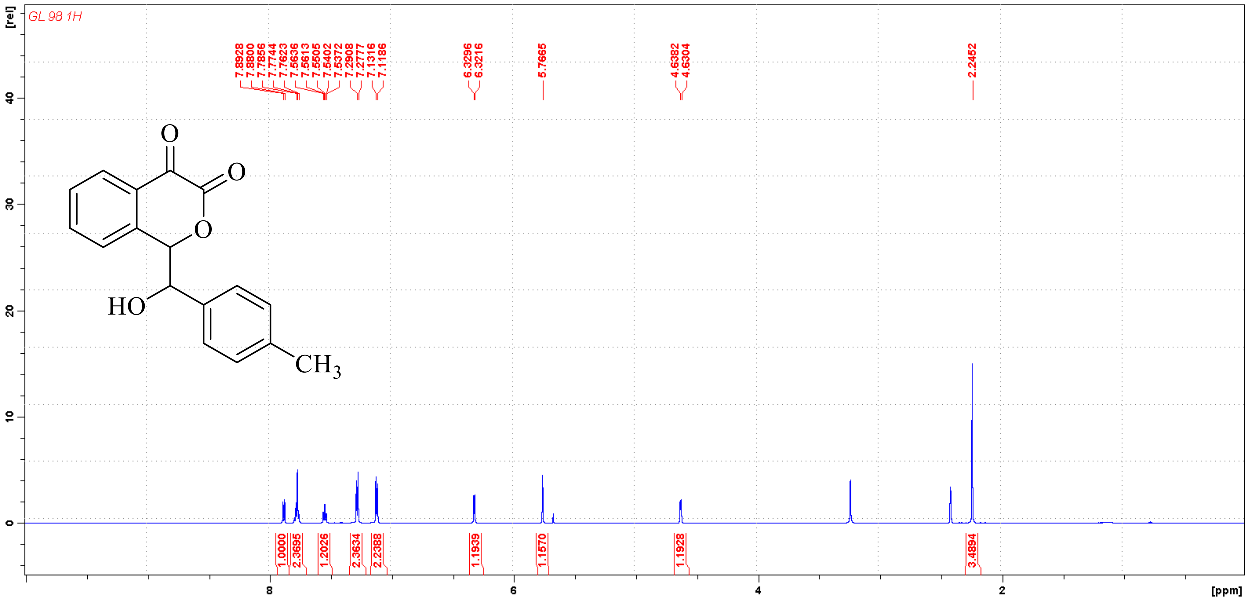


$^{13}$C{$^1$H} NMR (150 MHz, DMSO-d6)

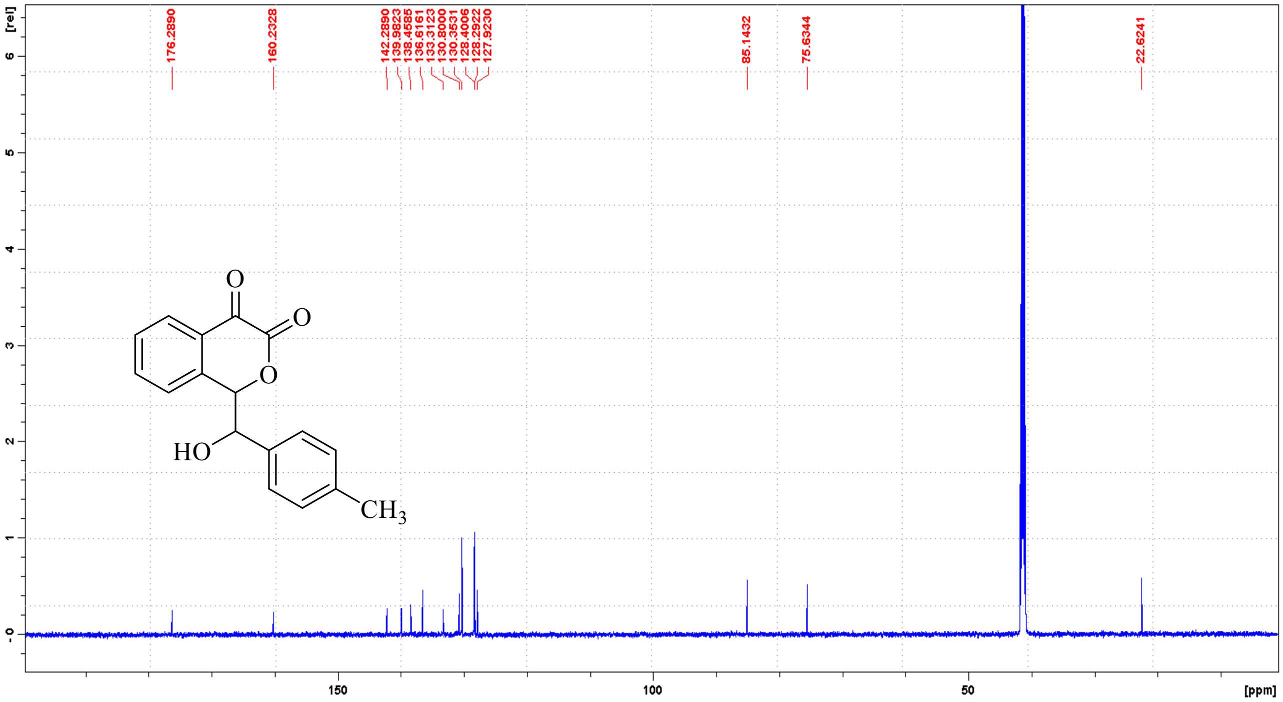

## 1-(hydroxy(o-tolyl)methyl)isochromane-3,4-dione Gl-146 (9n)

$^{1}H$ NMR (300 MHz, $CDCl_3$)

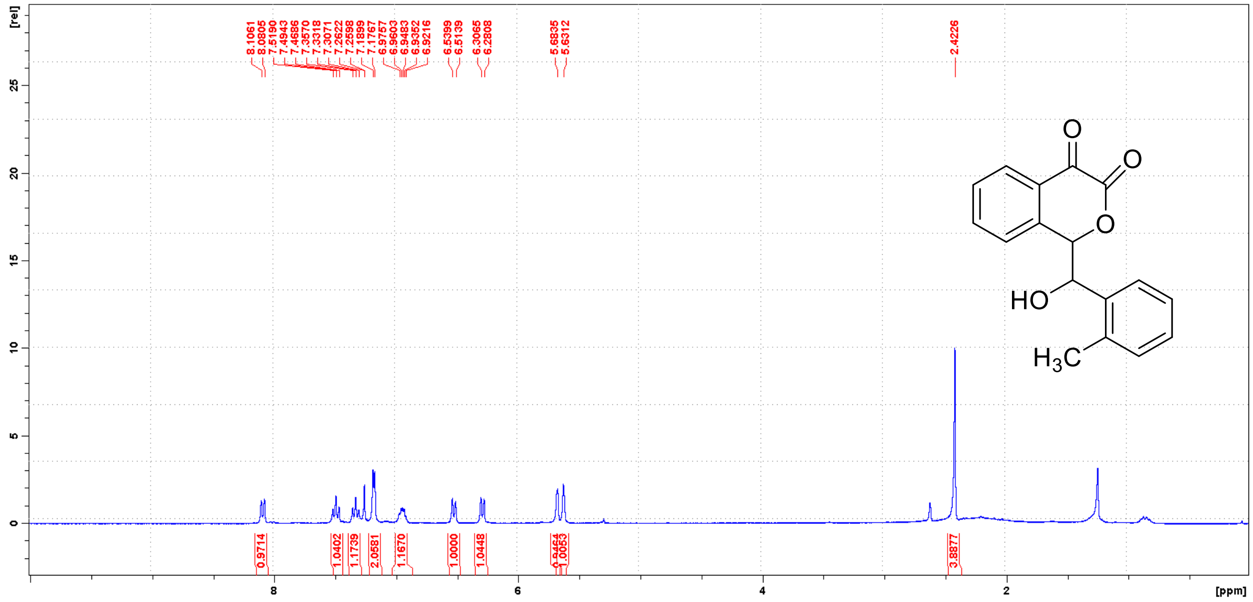

$^{13}C\{^{1}H\}$ NMR (75 MHz, $CDCl_3$)

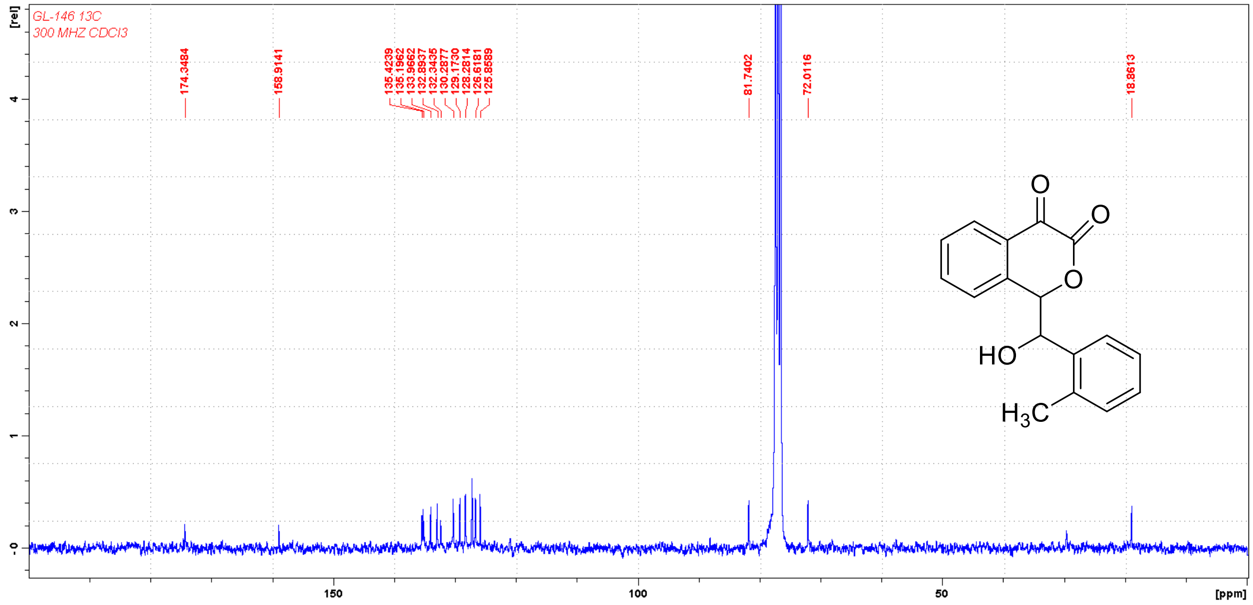

**1-(1-hydroxyoctyl)isochromane-3,4-dione (9o)**

$^{1}$H NMR (400 MHz, DMSO-d6)

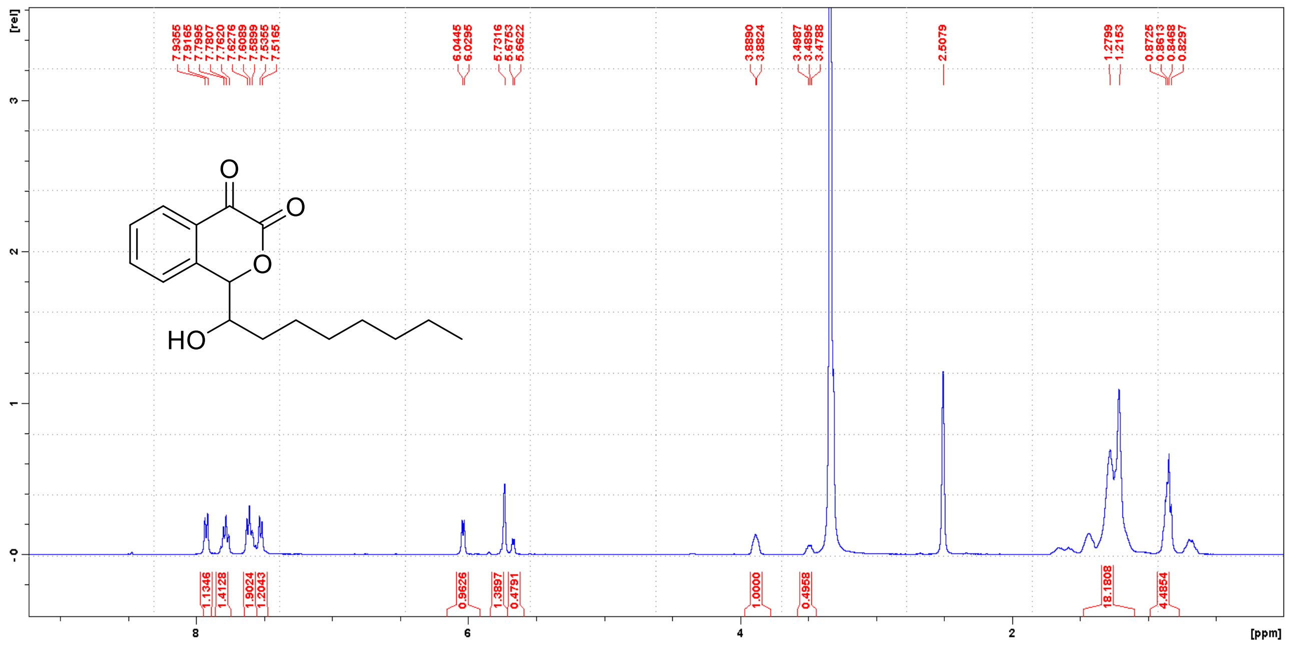

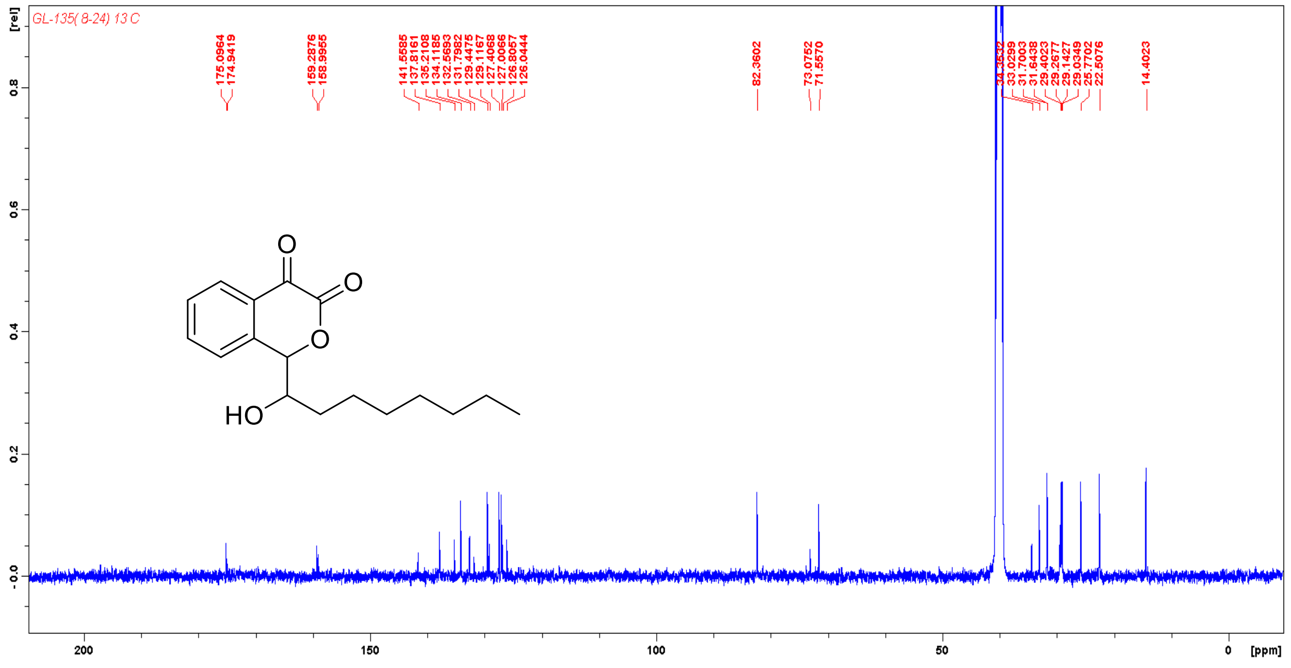

## 1-(1hydroxydecyl) isochromanone-3,4-dione (9p)

$^{1}$H NMR (300 MHz, DMSO-d6)

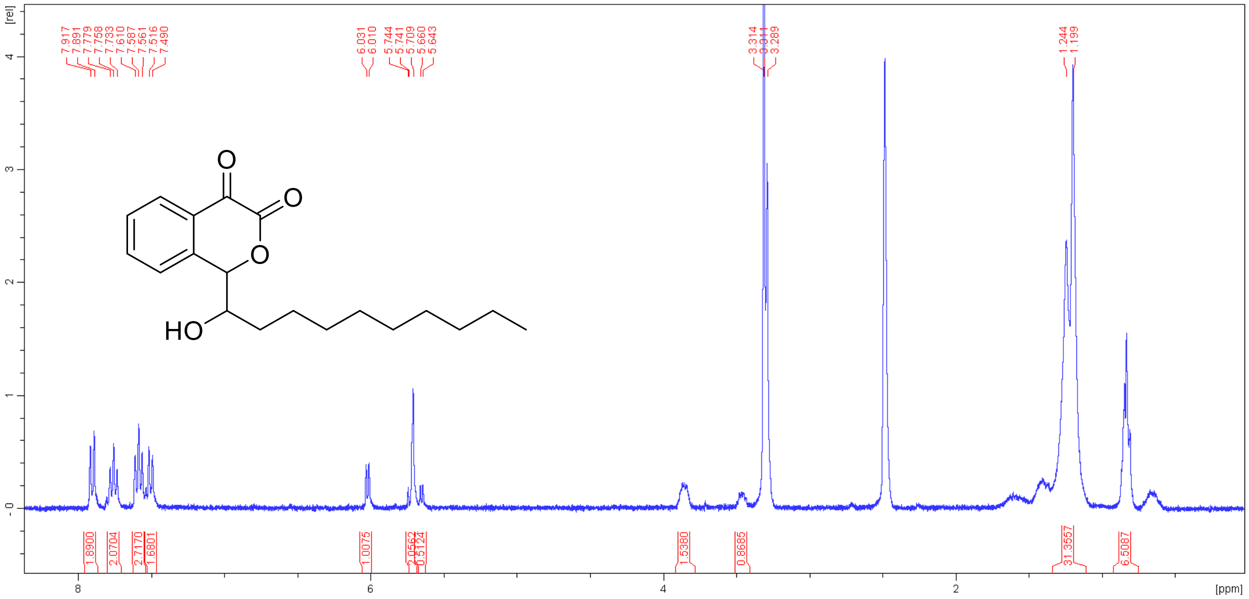


$^{13}$C{$^{1}$H} NMR (100 MHz, DMSO-d6)

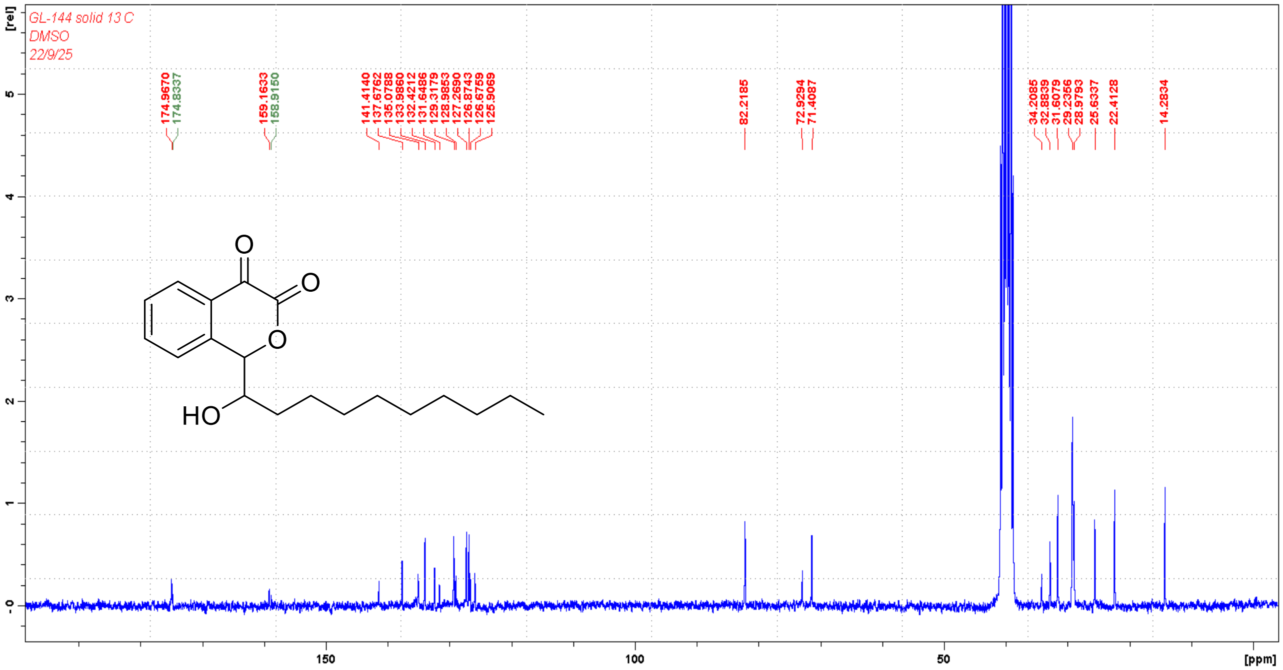

**1-(hydroxy(2-ioddophenyl)methyl)-6-methoxyisochromane-3,4-dione (9q)**

$^{1}$H NMR (300 MHz, DMSO-d6)

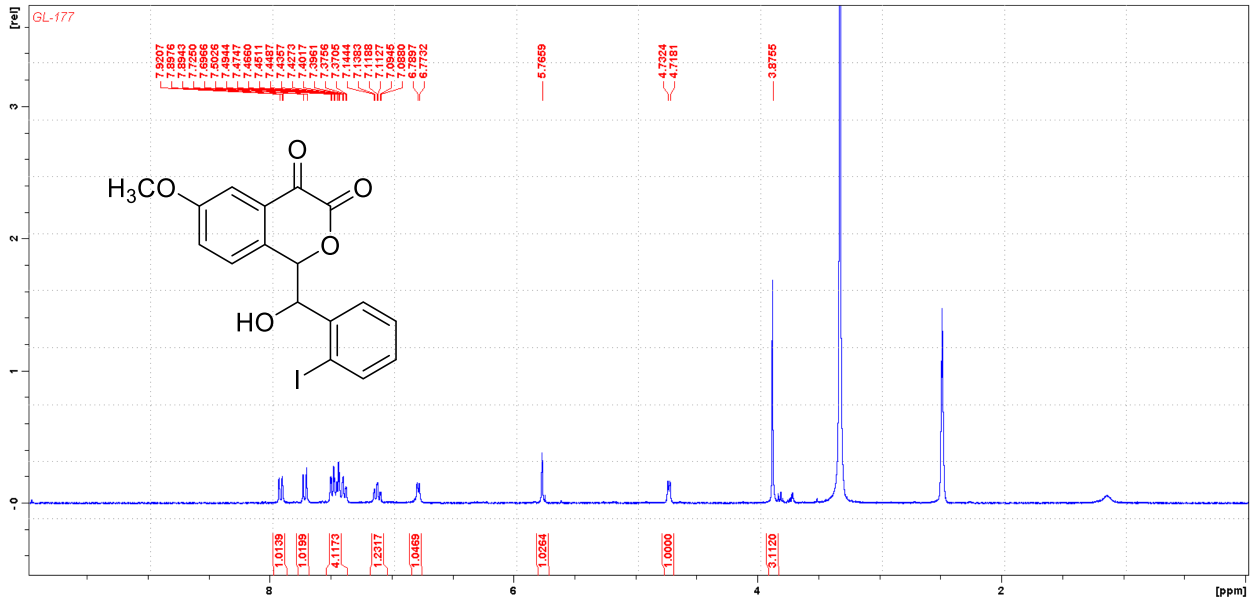

$^{13}$C{$^{1}$H} NMR (63 MHz, DMSO-d6)

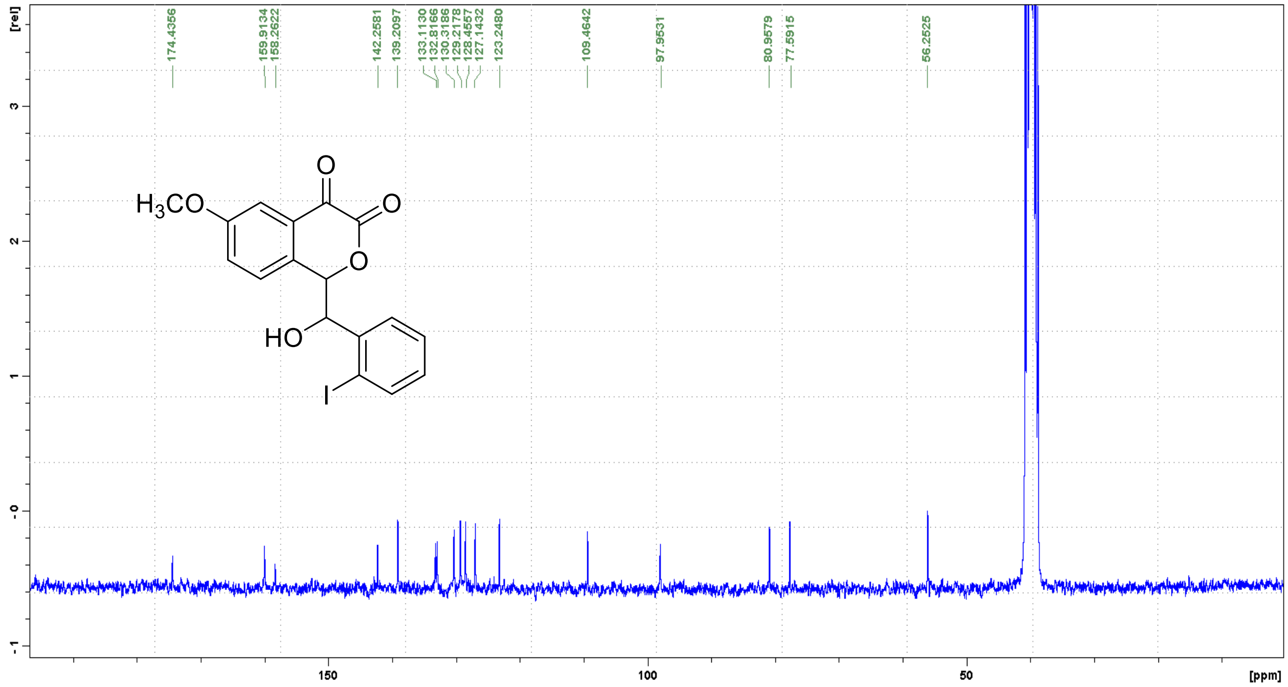

## 7-Bromo-1-(hydroxy(4-nitrophenyl) methyl)isochromanone (9r)

$^{1}$H NMR (300 MHz, DMSO-d6)

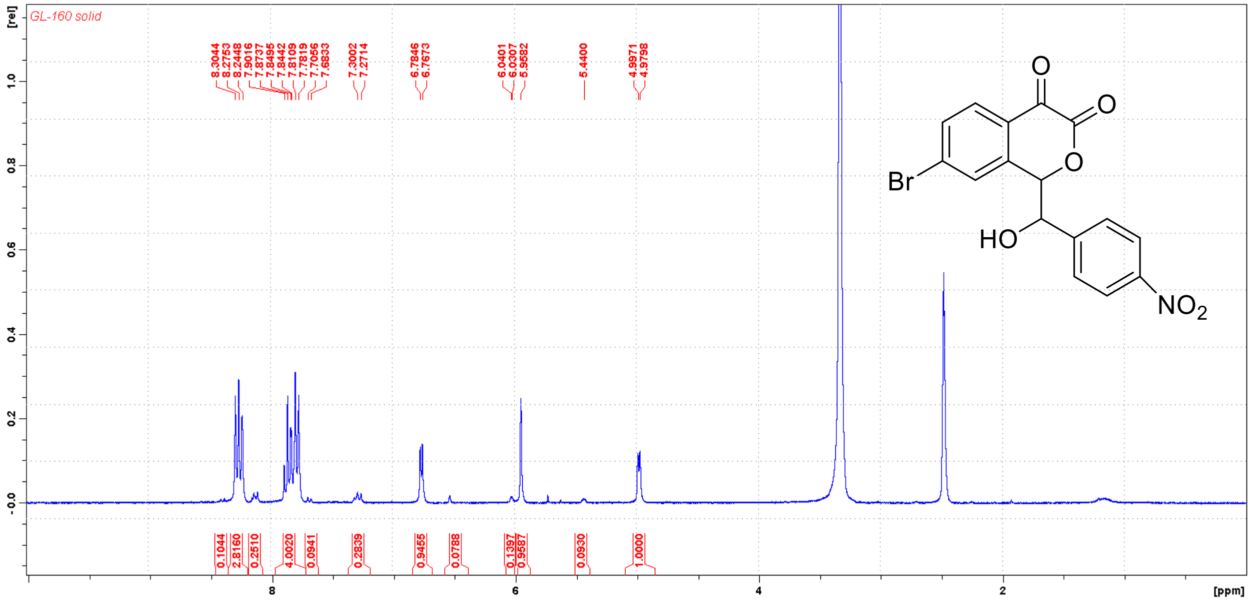

$^{13}$C{$^{1}$H} NMR (63 MHz, DMSO-d6)

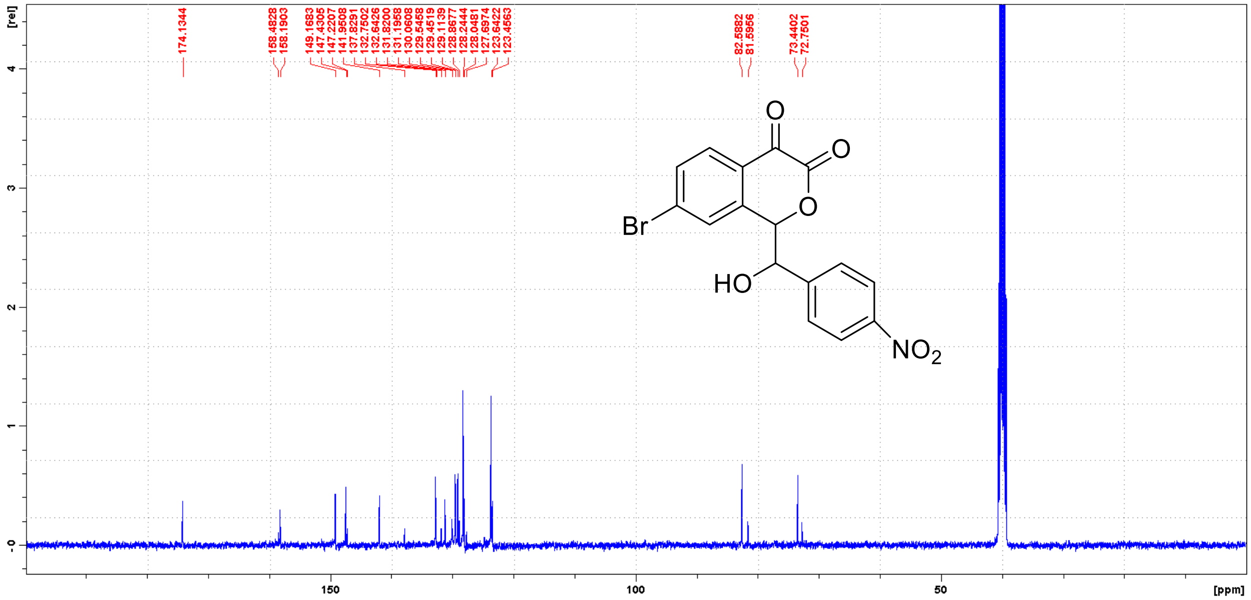

**1-(hyddroxy(4-nitrophenyl)methyl)-6-methoxyisochromanone-3,4-dione (9s)**

$^{1}$H NMR (400 MHz, DMSO-d6)

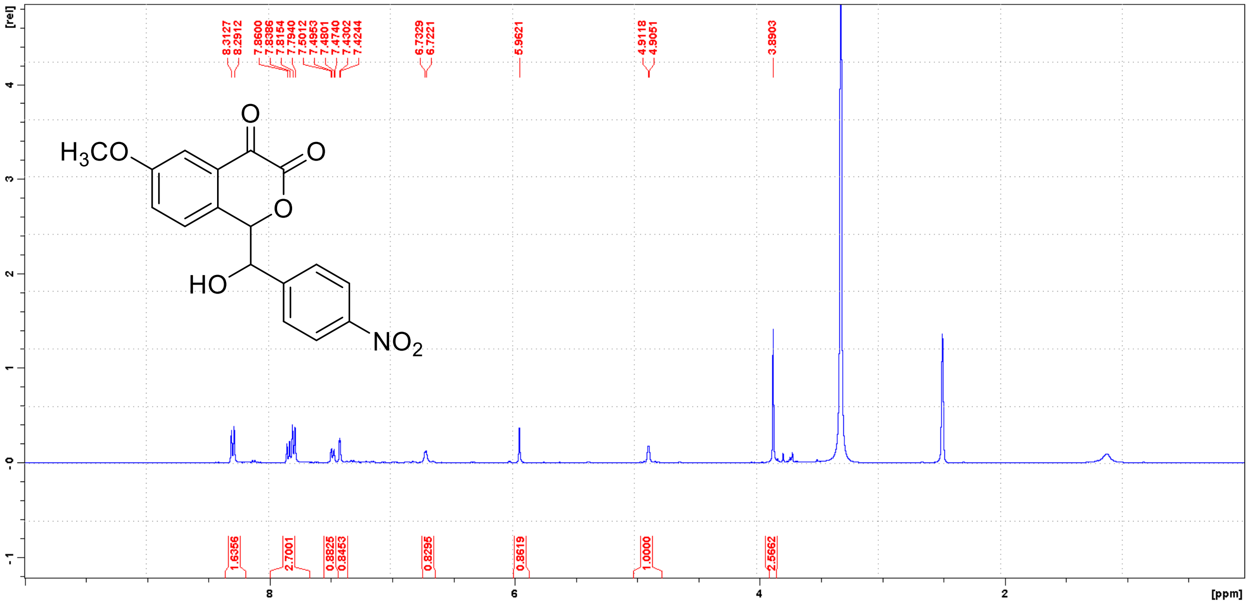


$^{13}$C{$^{1}$H} NMR (100 MHz, DMSO-d6)

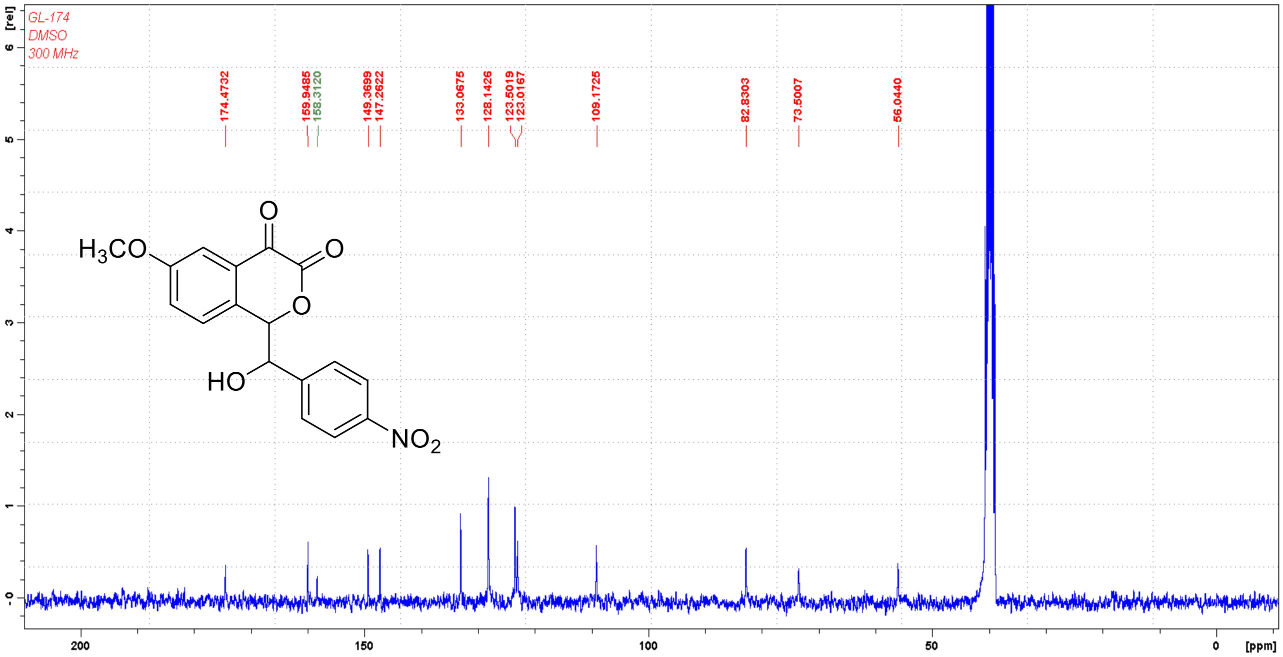

**1-((3-bromophenyl)(hydroxy)methyl)-8-nitroisochromanone-3,4-dione (9t)**

$^{1}H$ NMR (400 MHz, DMSO-d6)

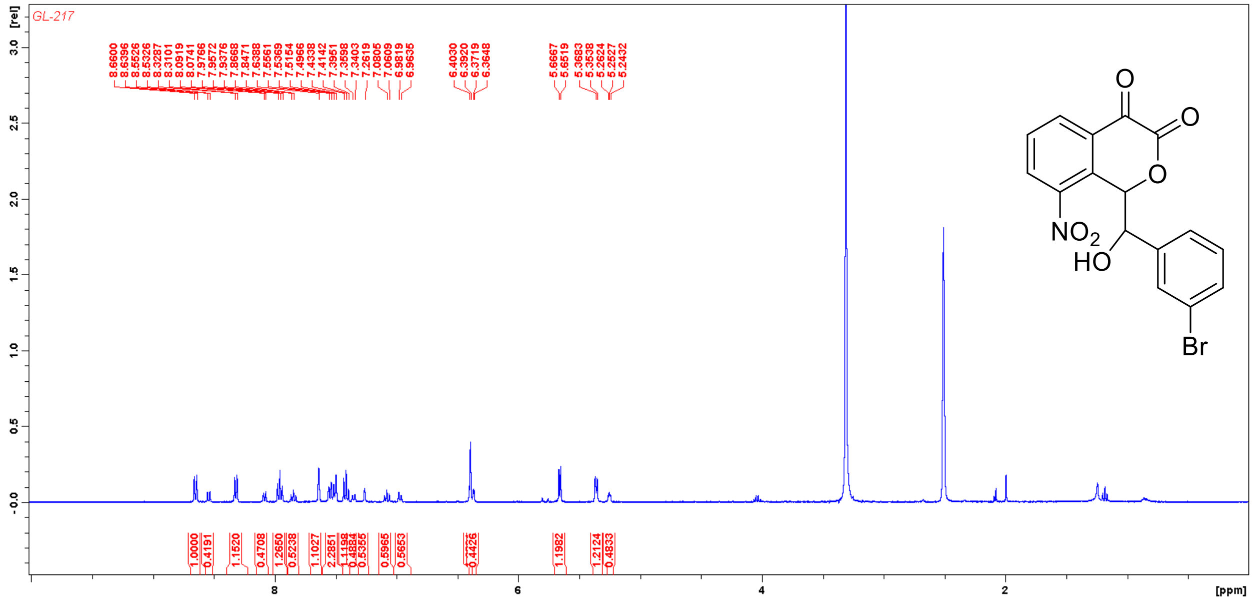


$^{13}C\{^{1}H\}$ NMR (100 MHz, DMSO-d6)

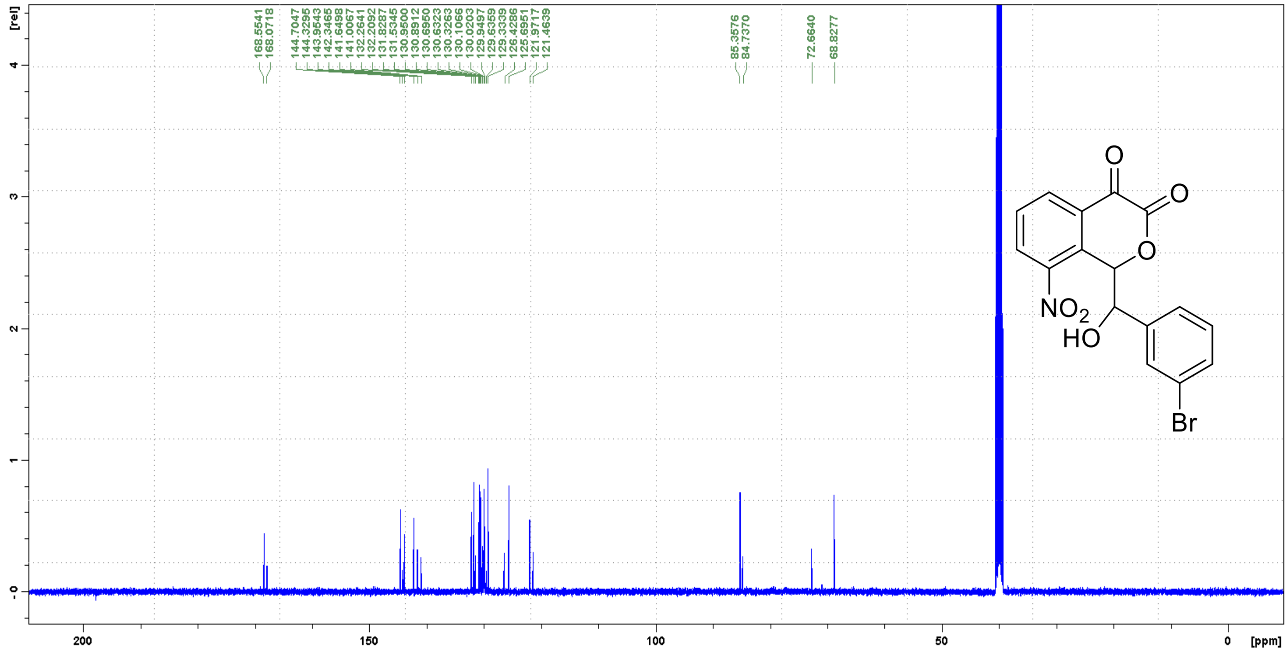

## 1-(2-(1,2-dihydroxyethyl)phenyl)-2-phenylethane-1,2-diol (10)

$^{1}$H NMR (600 MHz, MeOD-d4)

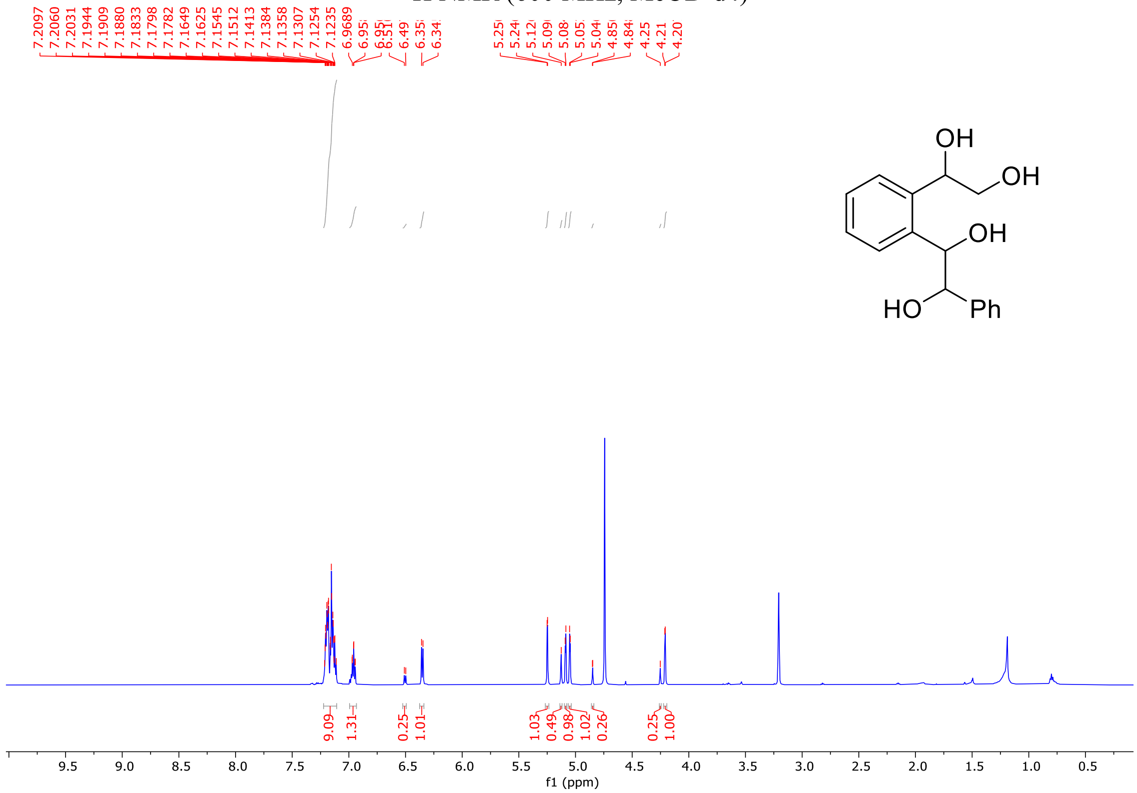

$^{13}$C{$^{1}$H} NMR (150 MHz, MeOD-d4)

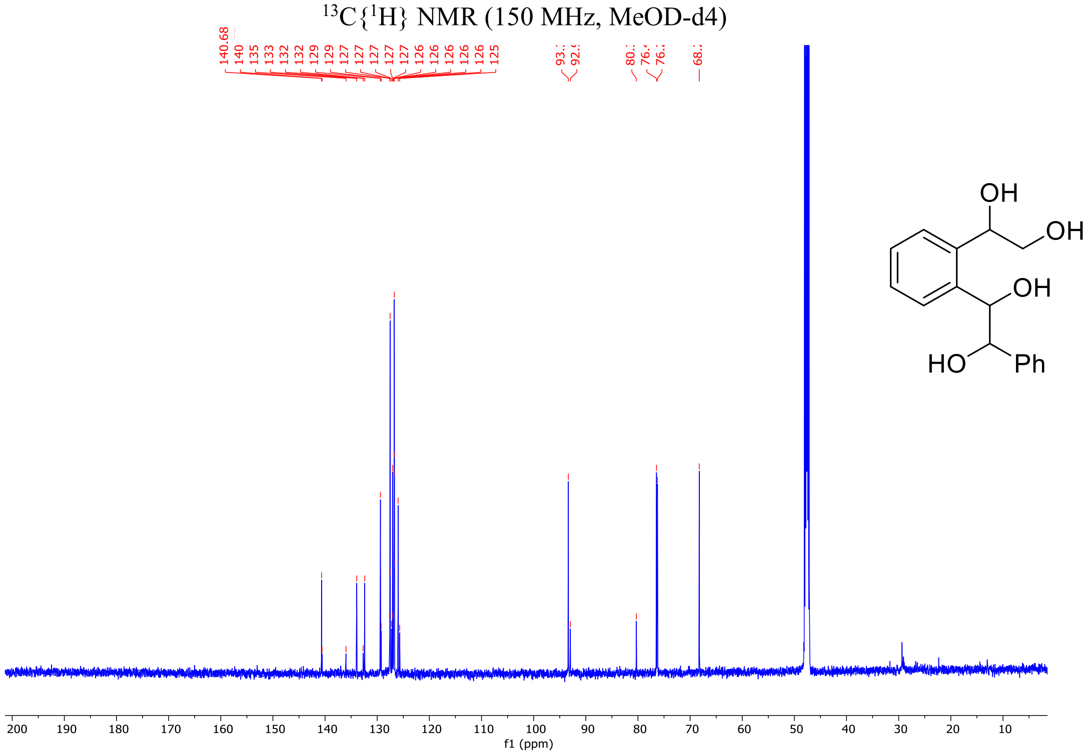

## References:

[1] Mousavi, M.S.; Di Mola, A.; Pierri, G.; Massa, A. Isochroman-3,4-dione and tandem aerobic oxidation of 4-bromoisochroman-3-one in the highly regio- and diastereoselective Diels–Alder reaction for the construction of bridged polycyclic lactones. *J. Org. Chem.* **2024**, *89*, 18602–18611. https://doi.org/10.1021/acs.joc.4c02522.

[2] Mousavi, M.S.; Tedesco, C.; Massa, A. Bifunctional organocatalysts and Brønsted bases in cooperative catalysis for the asymmetric [4+2] cycloaddition of in situ-generated dienes from isochromane-3,4-diones with nitroalkenes. *Adv. Synth. Catal.* **2025**, *367*, e70039. https://doi.org/10.1002/adsc.70039.

[3] Mousavi, M.S.; Massa, A.; Waser, M. Syntheses of 1,2,3-functionalized naphthols and phenols by decarboxylative cycloaddition/aromatization reactions of α-oxygenated lactones with allenoates or electron-deficient alkynes. *Org. Chem. Front.,* **2026***, 13,* 1573-1577 DOI: 10.1039/D5QO01582K.

[4] APEX3, version 2015.5-2; Bruker AXS Inc.: Madison, WI, USA, **2016**.

[5] SAINT, version 8.34A; Bruker AXS Inc.: Madison, WI, USA, **2013**.

[6] Sheldrick, G.M. SADABS, version 2014/5; Bruker AXS Inc.: Madison, WI, USA, **2014**.

[7] Sheldrick, G.M. A short history of SHELX. *Acta Crystallogr.* **2008**, 64, 112–122.

[8] Sheldrick, G.M. Crystal structure refinement with SHELXL. *Acta Crystallogr.* **2015**, 71, 3–8.

[9] Dolomanov, O.V.; Bourhis, L.J.; Gildea, R.J.; Howard, J.A.K.; Puschmann, H. OLEX2: A complete structure solution, refinement and analysis program. *Acta Crystallogr.* **2009**, 42, 339–341.
